# Metal tolerance gene family in barley: an in silico comprehensive analysis

**DOI:** 10.1007/s13353-022-00744-6

**Published:** 2022-12-31

**Authors:** Umesh Kumar Tanwar, Ewelina Stolarska, Elżbieta Rudy, Ewelina Paluch-Lubawa, Magda Grabsztunowicz, Magdalena Arasimowicz-Jelonek, Ewa Sobieszczuk-Nowicka

**Affiliations:** 1 Department of Plant Physiology, Faculty of Biology, Adam Mickiewicz University, ul. Uniwersytetu Poznańskiego 6, 61-614 Poznań, Poland; 2 Department of Plant Ecophysiology, Faculty of Biology, Adam Mickiewicz University, ul. Uniwersytetu Poznańskiego 6, 61-614 Poznań, Poland

**Keywords:** Metal-tolerance proteins, Barley, Metal stress, Gene expression

## Abstract

Metal-tolerance proteins (MTPs) are divalent cation transporters that play critical roles in metal tolerance and ion homeostasis in plants. However, a comprehensive study of MTPs is still lacking in crop plants. The current study aimed to comprehensively identify and characterize the *MTP* gene family in barley (*Hordeum vulgare*, *Hv*), an important crop. In total, 12 *HvMTPs* were identified in the barley genome in this study. They were divided into three phylogenetic groups (Zn-cation diffusion facilitator proteins [CDFs], Fe/Zn-CDFs, and Mn-CDFs) and further subdivided into seven groups (G1, G5, G6, G7, G8, G9, and G12). The majority of MTPs were hydrophobic proteins found in the vacuolar membrane. Gene duplication analysis of *HvMTPs* revealed one pair of segmental-like duplications in the barley genome. Evolutionary analysis suggested that barley *MTPs* underwent purifying natural selection. Additionally, the *HvMTPs* were analyzed in the pan-genome sequences of barley (20 accessions), which suggests that *HvMTPs* are highly conserved in barley evolution. *Cis*-acting regulatory elements, microRNA target sites, and protein–protein interaction analysis indicated the role of HvMTPs in a variety of biological processes. Expression profiling suggests that *HvMTPs* play an active role in maintaining barley nutrient homeostasis throughout its life cycle, and their expression levels were not significantly altered by abiotic stresses like cold, drought, or heat. The expression of barley *HvMTP* genes in the presence of heavy metals such as Zn^2+^, Cu^2+^, As^3+^, and Cd^2+^ revealed that these *MTPs* were induced by at least one metal ion, implying their involvement in metal tolerance or transportation. The identification and comprehensive investigation of *MTP* gene family members will provide important gene resources for the genetic improvement of crops for metal tolerance, bioremediation, or biofortification of staple crops.

**Supplementary Information:**

The online version contains supplementary material available at 10.1007/s13353-022-00744-6.

## Introduction

Heavy metals, which may be found in soil, act as co-factors, being crucial components required for optimal biological and plant cell growth (Thomine and Vert 2013). Divalent cations, like copper (Cu^2+^), iron (Fe^2+^), zinc (Zn^2+^), manganese (Mn^2+^), and nickel (Ni^2+^), are such essential trace elements for plants. These ions are necessary for the proper functioning of physiological processes; however, when in excess, they can induce toxic effects (Kolaj-Robin et al. 2015). Among others, the processes for which the presence of these ions is vital are protein processing, photosynthesis, and replication of DNA as well as electron transport in mitochondria and/or chloroplasts. The low level of these ions in plant tissues can also be detrimental, affecting growth and development (Bhardwaj et al. 2020). Due to the need to precisely maintain the appropriate level of metal ions, plants have developed a complex machinery allowing them to control the uptake, trafficking, mobilization, translocation, efflux, and storage of essential metal ions (Liu et al. 2019). To do so, a variety of transporters can be found in plants, such as the natural resistance-associated macrophage proteins (NRAMP), cation exchanger (CAX) family, or cation diffusion facilitator (CDF) proteins (Jiang et al. 2022). In our study, we focused on CDFs which are divalent cation (Zn^2+^, Co^2+^, Fe^2+^, Cd^2+^, Ni^2+^, and Mn^2+^) transporters that play crucial roles in metal homeostasis.

The CDF transporters in plants are denoted as metal-tolerance proteins (MTPs) (Lang et al. 2011; Ricachenevsky et al. 2013). They use an antiport mechanism, transporting divalent cations in exchange for H^+^, thus mediating metal homeostasis and tolerance controlling the outflow from the cytoplasm (Fu et al. 2017). MTPs are categorized into three different classes Mn-CDFs, Fe/Zn-CDFs, and Zn-CDFs, which is based on the substrate specificity (Montanini et al. 2007). Those three families can be further divided into seven groups based on the phylogenetic relationship and annotations conducted in *Arabidopsis thaliana* (Gustin et al. 2011). Based on this classification, groups 1, 5, and 12 are Zn-CDFs; groups 6 and 7 are Fe/Zn-CDFs, and 8 and 9 are the Mn-CDFs (Gustin et al. 2011). In two model plant species, *A. thaliana* and *Oryza sativa*, 12 and 10 *MTP* genes, respectively, were identified at first (Montanini et al. 2007). Since then some of them have been functionally characterized. These MTP proteins have been reported to transport a variety of metallic elements, including Fe, Mn, Cu, Zn, Ni, Co, and Cd (Das et al. 2021). For instance, AtMTP1 and AtMTP3 have been shown to localize in the tonoplast in *Arabidopsis*, conferring Zn and/or Co tolerance by sequestering excess Zn^2+^ and/or Co^2+^ (Arrivault et al. 2006; Kobae et al. 2004). AtMTP12 interacts with AtMTP5 to form a functional complex that transports Zn^2+^ from the cytosol to the Golgi apparatus (Fujiwara et al. 2015). MTP8 is one of the most extensively studied Mn-CDF family members, contributing to Mn detoxification in *A. thaliana* and *O. sativa* by sequestering Mn in vacuoles (Chen et al. 2013; Eroglu et al. 2016; Takemoto et al. 2017). *MTP* gene family members have already been identified in several plant species; however, *MTPs* in *A. thaliana* have received much attention (Eroglu 2015; Eroglu et al. 2016; Peiter et al. 2007). In monocots, most of the characterized members of *MTPs* belong to rice (Chen et al. 2013; Ma et al. 2018; Menguer et al. 2013; Montanini et al. 2007; Takemoto et al. 2017; Tsunemitsu et al. 2018; Ueno et al. 2015). Therefore, a thorough understanding of *MTPs* in other important crop plants is of great interest.

Barley (*Hordeum vulgare*) is a metal-tolerant crop, tolerating Mn (Foy et al. 1988), Cu, and Cd (González et al. 2017; Gvozdenac et al. 2013). As it is a member of the grass family, *H. vulgare* is one of the most important crops, being the fourth most cultivated worldwide among grain cereals after maize, wheat, and rice (Monat et al. 2019; Tanwar et al. 2022). The estimated production of barley for the year 2020–2021 was 159.74 million metric tons (https://www.statista.com/statistics/271973/world-barley-production-since-2008/). Barley is a diploid with a genome comprised of seven chromosomes (2*n* = 2*x* = 14) and an estimated size of 5.1 Gbp, with 80% of its genome composed of repetitive elements (Wicker et al. 2017). Despite such a large genome, barley is a very convenient genetic model for *Triticum aestivum*, which is hexaploid, due to the simpler diploid one. Additionally, many genes in barley and wheat possess corresponding and similar functions. Therefore, the knowledge gained in barley can be swiftly utilized to predict the genes with comparable functions in wheat (Monat et al. 2019). All of this makes barley unique among crop plants as it is tremendously important for agriculture and science.

Recent advancements in genomics have enabled the generation of large-scale sequence data for many crop species (Henry 2022; Varshney et al. 2015). Such datasets have aided in the study of genomic architecture and dynamics, as well as gene discovery. As genome sequences for more plant species become available, several MTP proteins have been identified genome-wide in many plant species, including *Vitis vinifera* (Shirazi et al. 2019), *Sorghum bicolor* (Ricachenevsky et al. 2013), *Brassica rapa (*Li et al. 2018*)*, *Nicotiana tabacum* (Liu et al. 2019), *Populus trichocarpa* (Gao et al. 2020), *Citrus sinensis* (Fu et al. 2017), and *Triticum aestivum* (Vatansever et al. 2017). Despite the fact that numerous genomic resources have been established to examine partial or entire genomic sequences and their related functions in barley, only *HvMTP8* has been studied in detail (Pedas et al. 2014). In recent years, efforts have been conducted to study the physio-biochemical and molecular mechanism of heavy metal tolerance in barley; however, *MTP* genes at the genome-scale in barley have not been well characterized until now.

To better understand the genome structure and fill the gaps in knowledge, we systematically explored the *MTPs* in barley to identify at the genome-scale level members of this family. In addition, we conducted phylogenetic relationships, gene structure, and protein motifs analysis as well as homology modeling. We also studied the expression patterns of identified *MTPs* in response to different heavy metal stresses. A such comprehensive investigation of *MTPs* genes in barley would establish the base to comprehend the role of transport proteins both in terms of their structure and physiological roles. The results of this study are not only a starting point for further functional research of the molecular mechanism(s) responsible for the tolerance of barley to heavy metals, but also allow for a better genetic understanding of this crop.

## Materials and methods

### Identification, phylogeny, and classification of *MTP *genes in barley

To identify the *MTP* genes in barley, the protein sequences of 12 MTPs in *Arabidopsis* obtained from Arabidopsis database (https://www.arabidopsis.org/) were used as a BLASTP query search with threshold *E*-value 1e^−10^ in *H. vulgare* Phytozome database (https://phytozome-next.jgi.doe.gov/). After removing the redundant sequences manually, the non-redundant sequences were examined with InterProScan (Finn et al. 2017), and the sequences possessing any of the typical domains of MTP proteins were recognized as MTP proteins. The barley *MTP* genes were named *HvMTP1.1* through *HvMTP12.1* as per the sequence homology obtained by phylogenetic analysis. Similarly, the *MTP* genes were identified in the barley pan-genome database downloaded from https://webblast.ipk-gatersleben.de/downloads/barley_pangenome/ (Jayakodi et al. 2020). The ratio of non-synonymous to synonymous substitution (Ka/Ks) of *MTP* homologs among the barley accessions was calculated by TBtools software (Chen et al. 2018). The physicochemical parameters of the identified MTP proteins in barley were computed using the ProtParam tool at the ExPASy resource portal (https://www.expasy.org/). Furthermore, the Plant-mPLoc web tool (http://www.csbio.sjtu.edu.cn/bioinf/plant-multi/) was used to predict the sub-cellular localizations, and TMHMM ServerV.2.0 and *HMMTOP* (https://services.healthtech.dtu.dk/service.php?TMHMM-2.0 and http://www.enzim.hu/hmmtop/html/submit.html, respectively) were used for the prediction of putative transmembrane regions.

For phylogenetic analysis, protein sequences of MTPs from various plants, including *A. thaliana*, *B. distachyon*, *O. sativa*, *P. trichocarpa*, *Z. mays*, *V. vinifera*, *S. bicolor*, and *C. sativus*, in addition to those of HvMTPs identified in this study were used. The ClustalW program with default settings was used for multiple sequence alignments of the protein sequences and the phylogenetic tree was generated by the MEGA-11 program (Tamura et al. 2021) using the neighbor-joining method with Poisson correction and 1000 bootstrap values. The phylogenetic tree was visualized on the iTOLv6 webtool (https://itol.embl.de/). Evolutionary analyses were conducted in MEGA-11 (Tamura et al. 2021), and the evolutionary distances were computed using the p-distance method.

### Gene structure, chromosomal localization, gene duplication, and syntenic gene analysis of *MTP* genes in barley

The gene structures of *HvMTP* genes were determined according to the genomic and CDS sequences by Gene Structure Display Serve2.0 software (http://gsds.gao-lab.org/). Chromosomal distributions of *HvMTP* genes were determined using the genome annotation (GFF3/GTF) files of barley available at the Ensembl Plant database (https://plants.ensembl.org/Hordeum_vulgare/Info/Index). The figures for exon/intron organization and chromosomal distributions were drawn by TBtools (Chen et al. 2018). Gene duplication analysis was carried out by using the Multiple Collinearity Scan toolkit (MCScanX) with the default settings (Wang et al. 2012), and the duplicated genes were visualized by TBtools software (Chen et al. 2018). The ratio of non-synonymous to synonymous substitution (Ka/Ks) was calculated by TBtools software. The divergence time (T) was estimated as *T* = Ks/(2 × 6.5 × 10^− 9^) × 10^−6^ MYA for monocots (Cui et al. 2019; Ju et al. 2019; Lynch and Conery 2000; Wolfe et al. 1989), based on a rate of 6.5 × 10^−9^ substitutions per site per year. To explore the synteny relationships of the orthologous *MTP* genes among barley and other species, genome data and the gene annotation files of *A. thaliana*, *O. sativa*, *B. distachyon*, and *S. bicolor* were downloaded from the Phytozome database (https://phytozome-next.jgi.doe.gov/). The syntenic analysis graphs were constructed by using the Dual Synteny Plotter function in TBtools software.

### Conserved motifs, domain architectures, and homology modeling of HvMTP proteins

The conserved motifs in the protein sequences of *HvMTP* genes were predicted by the MEME suite 5.3.3 (https://meme-suite.org/meme/tools/meme) based on “zero or one occurrence per sequence (zoops)”. The HMMER database by the Inter-pro scan program (http://www.ebi.ac.uk/interpro/) was used for the Hidden Markov Model analysis. The NCBI-Conserved Domains Database (CDD) tool was used to identify the typical domains of MTP in HvMTP proteins. The conserved motifs and domain architectures were visualized by TBtools. To predict the secondary structure of HvMTP proteins, SOPMA (https://npsa-prabi.ibcp.fr/cgi192bin/npsa_automat.pl?page=/NPSA/npsa_sopma.html) was used with default parameters. *N*-glycosylation sites of HvMTP proteins were predicted by NetNGlyc-1.0-services (https://services.healthtech.dtu.dk/service.php?NetNGlyc-1.0). Further, the 3D structures of HvMTP proteins were predicted by the homology modeling method. First, the position-specific iterated BLAST algorithm (PSI-BLAST) was utilized to find the most similar homology in the PDB database (http://www.rcsb.org/), and then the Swiss-Model interactive tool (https://swissmodel.expasy.org/interactive/) was used to predict the 3D structure of the HvMTP proteins. Additionally, the PROCHECK test was used to inspect the 3D structure of MTP protein in the SAVES server (http://nihserver.mbi.ucla.edu/SAVES/).

### *Cis*-acting regulatory elements, microRNA target sites, and protein–protein interaction analysis of *HvMTP* genes

The sequences of the 1500 bp upstream region of the start codons of *HvMTP* genes were downloaded from the barley genome database and were analyzed by PlantCARE online tool (http://bioinformatics.psb.ugent.be/webtools/plantcare/html/) for *cis*-acting regulatory elements (CREs) analysis (Rombauts et al. 1999). The coding sequences of barley *MTP* genes were analyzed by psRNATarget server (https://www.zhaolab.org/psRNATarget/) for miRNA target site prediction (Dai et al. 2018). The protein–protein interaction analysis was carried out on the STRING web tool (https://string-db.org/) (Szklarczyk et al. 2021), and clustering was done as per k-means clustering.

### Expression analysis of *HvMTP* genes based on RNA-Seq data

The expression analysis of *HvMTP* genes in various developmental tissues of the barley plant, under abiotic stress, and heavy metal toxicity was carried out using publically available databases, Affymetrix Barley Genome Array and mRNA-Seq Gene Level *Hordeum vulgare* (ref: Morex V3) on GENEVESTIGATOR v3 tool (Grennan 2006). The heatmaps of gene expression data thus obtained were generated by using the TBtools software (Chen et al. 2018; Hruz et al. 2008).

## Results

### Identification, phylogeny, and classification of *MTP* genes in barley

**Fig. 1:**
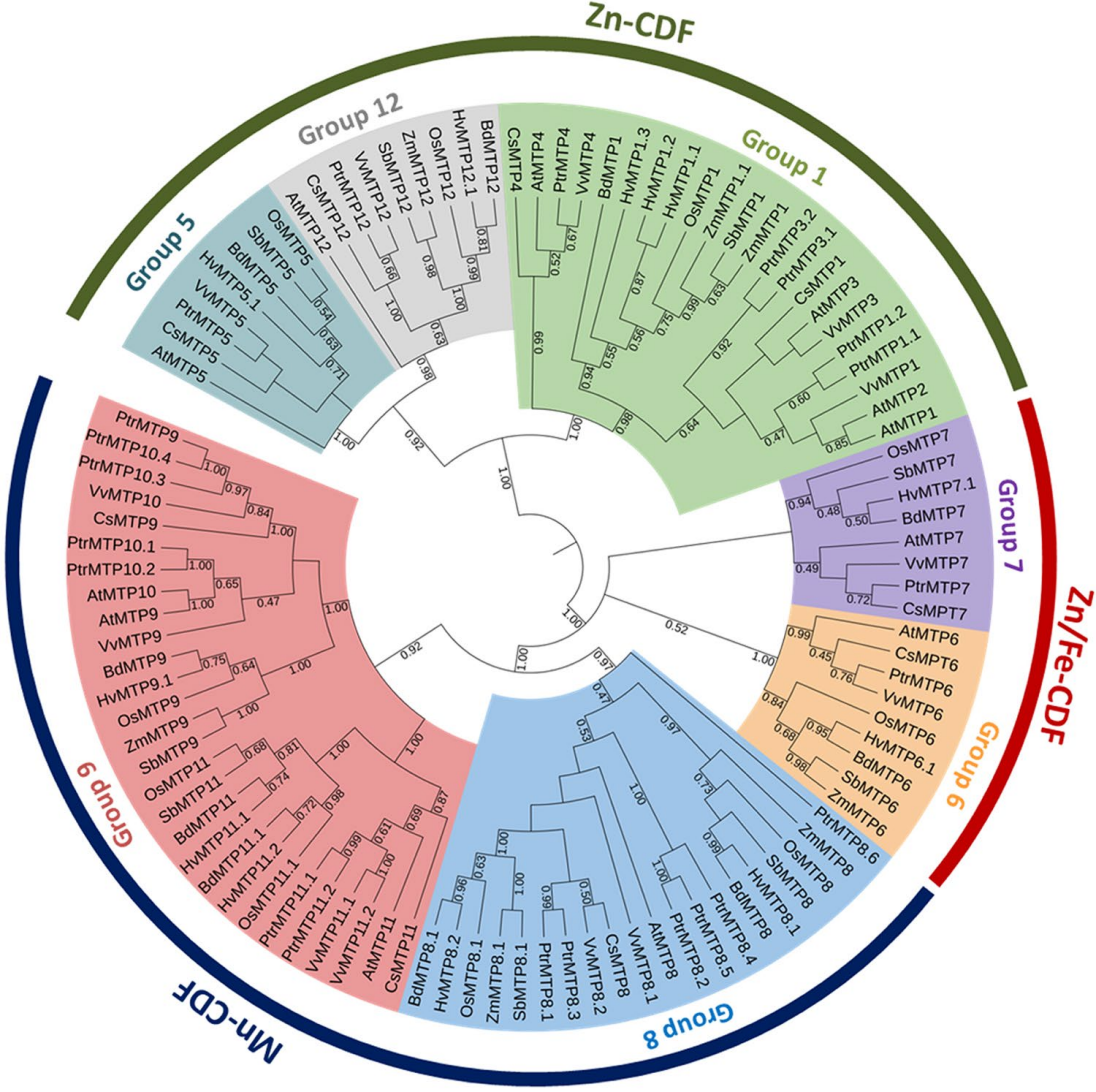
Phylogenetic relationship of MTP proteins in *H. vulgare (Hv)* and other plants, *A. thaliana* (*At*), *B. distachyon* (*Bd*), *O. sativa* (*Os*), *P. trichocarpa* (*Ptr*), *Z. mays* (*Zm*), *V. vinifera* (*Vv*), *S. bicolor* (*Sb*), *C. sativus* (*Cs*)*.* The evolutionary relationships were constructed using the neighbor-joining (NJ) method with 1000 bootstrap replications by MEGA-11 software. All 104 MTP proteins are clustered into three major substrate-specific groups and seven primary groups which are highlighted in different colors. *MTP* metal-tolerance protein

By using 12 AtMTP protein sequences from *Arabidopsis* as the query, a total of 12 *HvMTP* genes were found in the genome of *H. vulgare*. The phylogenetic relationship and sequence similarity of the HvMTPs with those of *A. thaliana* were investigated further. The 12 HvMTP proteins were named HvMTP1.1 to HvMTP12.1 based on their sequence similarity/cover values and orthologous relationship (Table 1). The evolutionary relationships of barley MTP proteins with 92 MTP protein sequences from representative monocot and dicot plant species were comprehensively analyzed, and a phylogenetic tree was constructed. These MTP proteins were classified into seven primary groups (1, 5, 6, 7, 8, 9, and 12), each of which belonged to one of three major substrate-specific groups (Zn-CDFs, Zn/Fe-CDFs, and Mn-CDFs; Fig. 1). Groups 1 and 9 are the largest of the seven groups, with three HvMTPs each, followed by group 8 with two HvMTPs. The rest of the groups 5, 6, 7, and 12 were the smallest groups with one HvMTP each. Interestingly, the barley pan-genome sequences analysis revealed that all 20 accessions of barley had 12 *MTP* genes (Supplementary Table S1). We further investigated the *MTP* homologs in the wild accession B1K-04–12 and other cultivated types, using Morex as a representative. The ratio of non-synonymous substitution (Ka) and synonymous substitution (Ks) was used to examine the selection pressure among *MTP* homologs. The Ka/Ks values for seven gene pairs were < 1, and the rest of the genes had zero/NA values (Supplementary Table S2).

**Table 1:** Detailed information of *HvMTP* genes identified in barley

Ensembl barley	Gene name	CDS length (bp)	Protein size (aa)	MW (kDa)	Theoretical pI	GRAVY	Sub-cellular localization	TMD
HORVU.MOREX.r3.2HG0174930.1	***HvMTP1.1***	1242	413	45.077	5.83	0.090	Tonoplast	6
HORVU.MOREX.r3.1HG0016110.1	***HvMTP1.2***	1266	421	45.830	5.95	0.053	Tonoplast	6
HORVU.MOREX.r3.4HG0409880.1	***HvMTP1.3***	1227	408	44.656	5.89	0.035	Tonoplast	6
HORVU.MOREX.r3.6HG0634070.1	***HvMTP5.1***	1089	362	39.650	8.54	0.231	Tonoplast	6
HORVU.MOREX.r3.4HG0376610.1	***HvMTP6.1***	1509	502	53.710	7.84	− 0.007	Tonoplast	5
HORVU.MOREX.r3.2HG0158870.1	***HvMTP7.1***	1386	461	50.016	7.28	0.044	Tonoplast	4
HORVU.MOREX.r3.6HG0617950.1	***HvMTP8.1***	1233	410	45.682	5.35	− 0.058	Tonoplast	4
HORVU.MOREX.r3.4HG0393950.1	***HvMTP8.2***	1203	400	44.879	5.27	0.069	Tonoplast	5
HORVU.MOREX.r3.3HG0222000.2	***HvMTP9.1***	1170	389	44.035	6.20	− 0.017	Cell membrane, tonoplast	6
HORVU.MOREX.r3.1HG0073140.1	***HvMTP11.2***	969	322	36.239	4.92	0.004	Tonoplast	3
HORVU.MOREX.r3.3HG0303160.1	***HvMTP11.1***	1200	399	44.687	5.34	0.057	Tonoplast	4
HORVU.MOREX.r3.2HG0119060.1	***HvMTP12.1***	2394	797	86.558	7.06	0.108	Nucleus, Tonoplast	11

*Hv H. vulgare*, *MTP* metal-tolerance protein, *CDS* coding sequence, *MW* molecular weight, *pI* isoelectric point, *GRAVY* grand average of hydropathy, *TMD* transmembrane domain

The characteristics of the *HvMTP* genes were also analyzed in detail (Table 1). The CDS length of *HvMTP* genes varied from 969 (*HvMTP11.2*) to 2394 bp (*HvMTP12.1*). The amino acid number of HvMTP proteins ranged from 322 (HvMTP11.2) to 797 (HvMTP12.1), and the molecular weight of HvMTP proteins varied from 36.23 (HvMTP11.2) to 86.55 kDa (HvMTP12.1). The GRAVY values of the HvMTPs ranged from − 0.058 (HvMTP8.1) to 0.231 (HvMTP5.1). The isoelectric point (pI) ranged from 4.92 (HvMTP11.2) to 8.54 (HvMTP5.1), with 7 HvMTP members pI < 7 and 5 HvMTP members pI > 7 (Table 1). The HvMTP proteins had a wide range of transmembrane domains (TMDs). Most of the HvMTPs contained 3–6 TMDs and HvMTP12.1 with 11 TMDs, respectively (Table 1 and Supplementary figure S1). Sub-cellular localization prediction showed that all the HvMTP proteins localized to the tonoplast, with dual localization predictions for HvMTP9.1 (cell membrane or tonoplast) and HvMTP12.1 (nucleus or tonoplast).

### Gene structure, chromosomal localization, gene duplication, and synteny analysis of *MTP* genes in barley

**Fig. 2:**
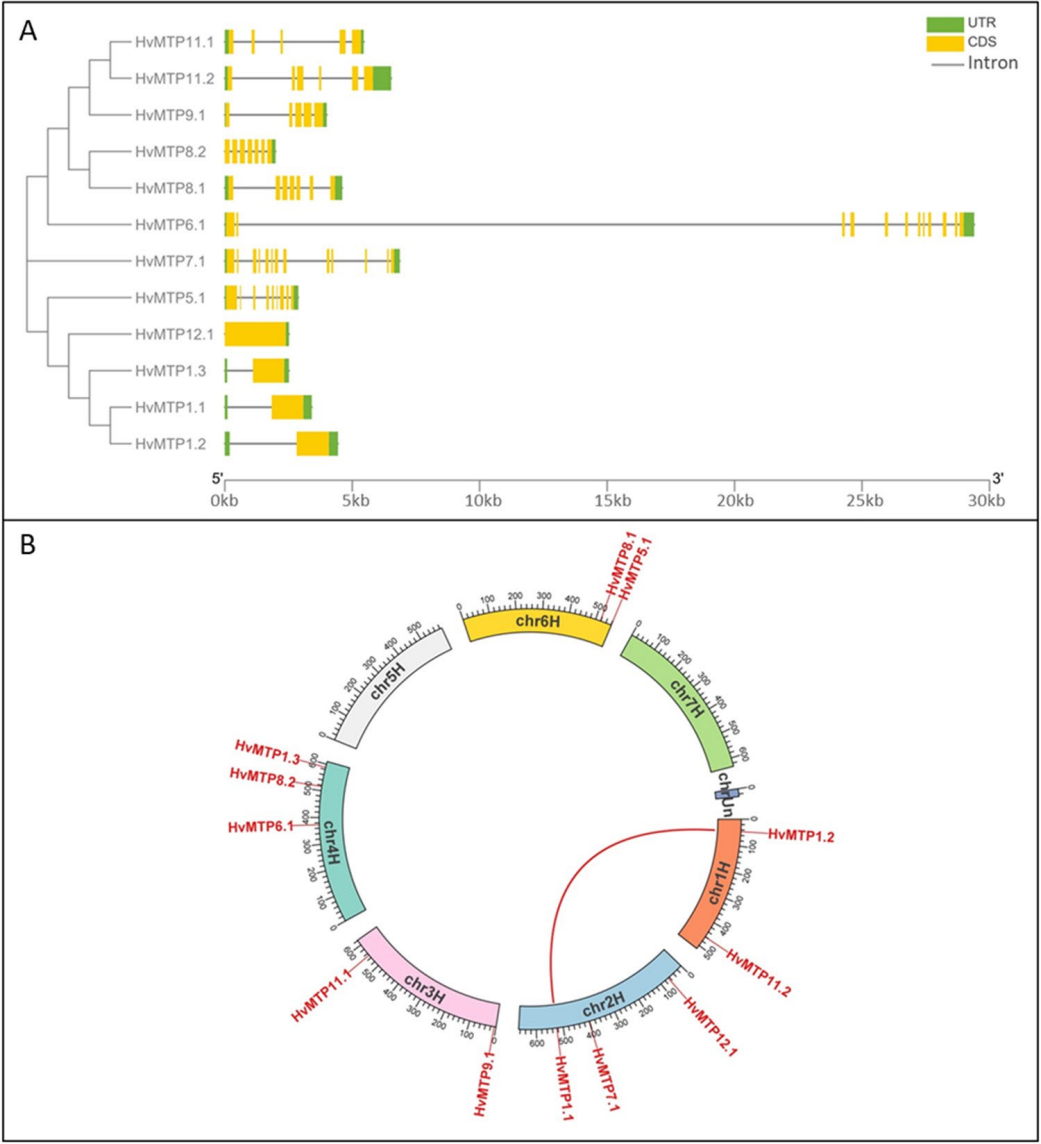
Gene structure (**A**) and the distributions of *HvMTP* genes on *H. vulgare* chromosomes (**B**). Chromosome number is indicated in black color, and gene names are in red. A segmental duplicated gene pair was linked with a red line. *Hv (H. vulgare)*; MTP (metal-tolerance protein); the scale bar in panel A shows gene length in kb (kilobase-pair)

We examined the exon–intron arrangements of the *HvMTP* genes. Exon–intron organizations for *HvMTP* genes belonging to the same groups were identical, as seen in Fig. 2A. Groups 1 and 12 of Zn-CDFs had only one exon (with or without an intron), whereas group 5 had nine exons. The Zn/Fe-CDF gene groups 6 and 7 had 12 and 13 exons, respectively. The Mn-CDFs in group 8 had seven exons, while most genes in group 9 had five exons, except *HvMTP11.2*, which had six exons.

*HvMTP* genes were located on 5 chromosomes out of seven chromosomes in barley (Fig. 2B). Chromosomes 4H and 2H contained the most genes as three genes each. Chromosomes 6H, 3H, and 1H contained two genes each, while no *HvMTP* gene was identified in chromosomes 5H and 7H. Most of the *HvMTP* genes that are located in the same chromosome showed greatly large distances. Gene duplication analysis of *HvMTP* genes revealed 1 segmental duplication gene pair in barley chromosomes. The duplicated genes (*HvMTP1.1/HvMTP1.2*) were located on chromosomes 2H and 1H (Fig. 2B). The ratio of non-synonymous substitution (Ka) and synonymous substitution (Ks) was used to examine the selection pressure among duplicated gene pairs. The Ka/Ks values for *HvMTP1.1/HvMTP1.2* gene pair was 0.132, and the estimated divergence time was about 11.142 MYA (Table 2).

**Table 2:** The ratio of non-synonymous to synonymous substitution analysis for duplicated *HvMTP* genes

Duplicated genes	Duplication type	Ka	Ks	Ka/Ks	MYA
*HvMTP1.1/HvMTP1.2*	Segmental	0.017968	0.135942	0.132174	11.14281178

*Hv H. vulgare*, *MTP* metal-tolerance protein

**Fig. 3:**
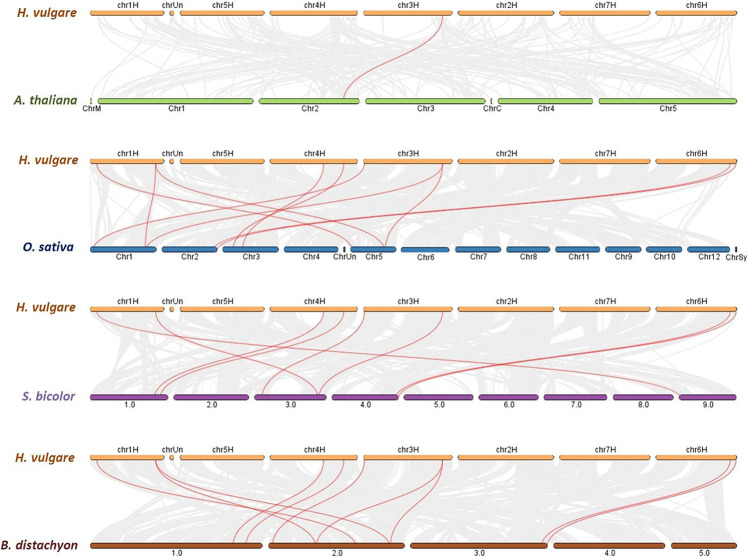
Synteny analysis of *MTP* genes in *H. vulgare* with other plant species, *A. thaliana*, *B. distachyon*, *O. sativa*, and *S. bicolor*. Gray lines in the background indicate the collinear blocks within *H. vulgare* and other plant genomes, while the red lines highlight the syntenic MTP gene pairs. MTP (metal-tolerance protein)

To further understand the evolutionary relationship of MTP family members, collinearity analyses were conducted between *H. vulgare*, *A. thaliana*, *B. distachyon*, *O. sativa*, and *S. bicolor* (Supplementary Table 3). A total of 8 HvMTPs (57.14%) were identified with collinearity relationship to MTPs in other monocot plant species, of which 8, 10, and 10 orthologous gene pairs were identified from *H. vulgare*-*S. bicolor*, *H. vulgare*-*B. distachyon*, and *H. vulgare*-*O. sativa*, respectively (Fig. 3). However, only one orthologous gene pair was detected from *H. vulgare*-*A. thaliana.*

### Conserved motifs, domain architectures, and homology modeling of HvMTP proteins

**Fig. 4:**
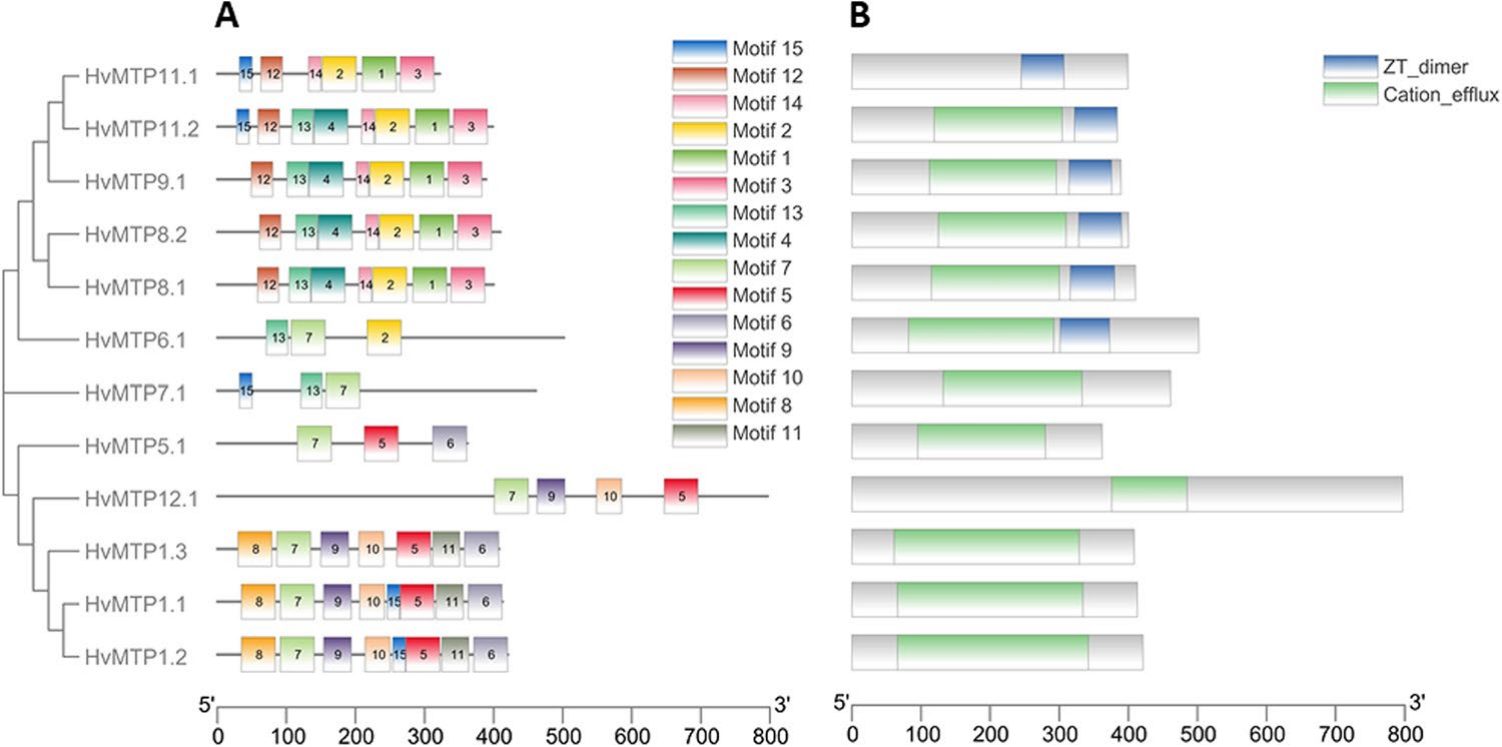
Distributions of the conserved motifs (**A**) and domains (**B**) in HvMTP proteins from barley. *Hv (H. vulgare)*; MTP (metal-tolerance protein)

A total of 15 conserved motifs were found in HvMTP proteins, five of which (motifs 2, 3, 4, 5, and 7) were recognized by InterProScan tools as being either TMDs or CTDs of cation efflux superfamilies (Fig. 4A and Supplementary Table 4). It was found that members of each cluster or group had a distinct distribution of conserved motifs (Fig. 4A). The Mn-CDF subfamily contained five conserved motifs (12, 14, 2, 1, and 3). Two motifs (13, 7) were conserved in the Fe/Zn-CDF subfamily, whereas three motifs (7, 5, and 6) were conserved in the Zn-CDF group.

All HvMTP proteins, with the exception of HvMTP11.1, were shown to include the MTP’s typical cation efflux domain, according to conserved domain analysis (Fig. 4B). Additionally, all members of the Mn-CDF cluster and one member (HvMTP6.1) of group 6 of the Fe/Zn-CDFs were shown to include the zinc transporter dimerization domain ZT-dimer (Fig. 4B). Furthermore, the highly conserved aspartic acid-based DxxxD (*X* = any amino acid) motifs and HxxxD motifs (*X* = any amino acid) with aspartic (D) and histidine (H) were also distributed differentially in three clusters of HvMTPs (Supplementary Figs. 2–4).

**Fig. 5:**
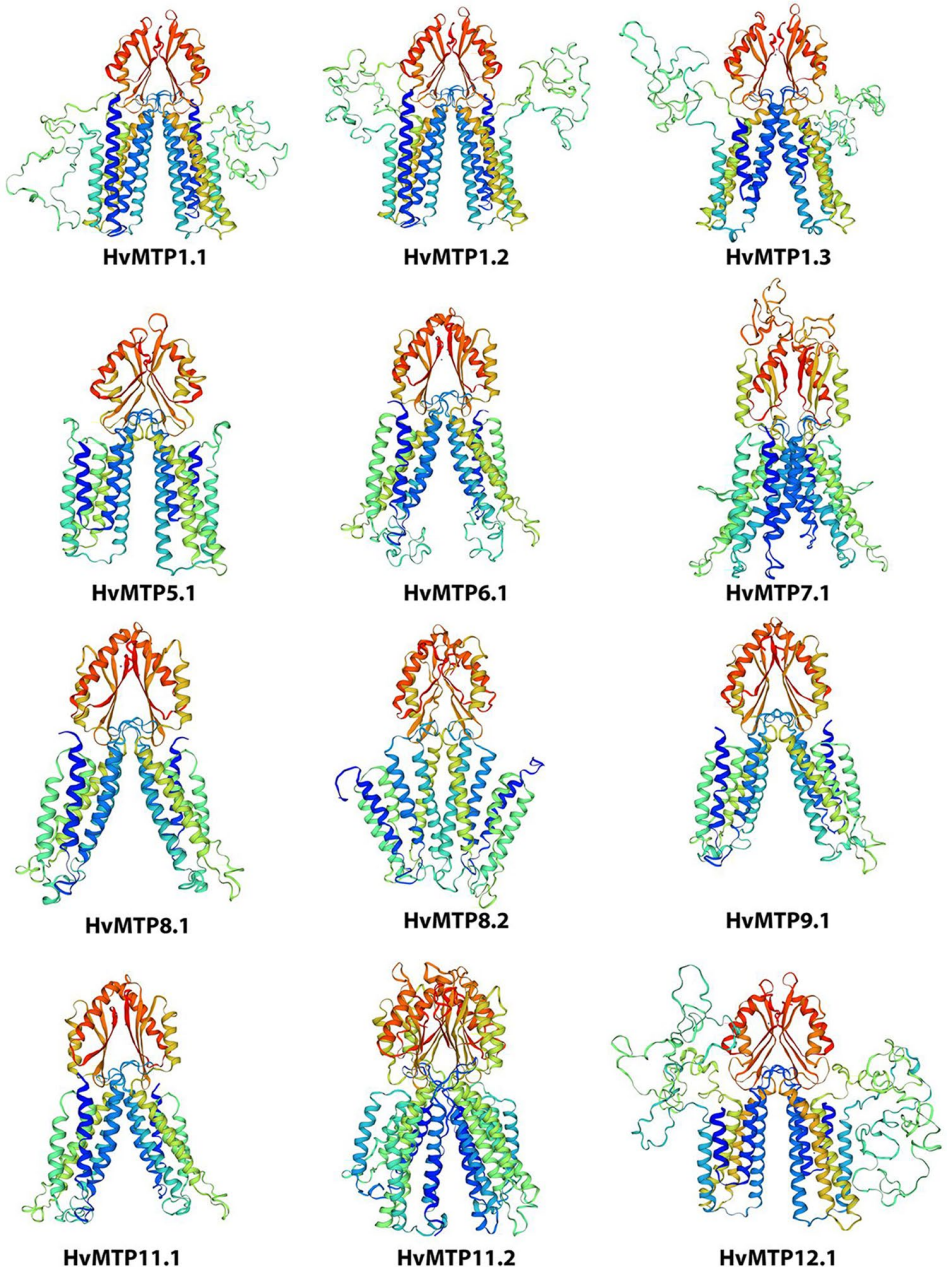
Predicted 3D structures of barley HvMTP proteins by Swiss-Model. Models were visualized by rainbow color from N to C terminus. The coils and the smooths represented alpha-helices and beta-sheets, respectively. *Hv (H. vulgare)*; MTP (metal-tolerance protein)

The Swiss-Model was used to model the sequences of HvMTP proteins in order to better understand their functions. The sequence identity of the 3D structure models ranged from 17.45 to 38.43%, the GMQE value ranged from 0.18 to 0.50, and the global score of QMEANDisCo ranged from 0.40 to 0.63 (Table 3). These findings indicated that the 3D protein structure models of HvMTP proteins were of high quality (Fig. 5). Only one subfamily of HvMTPs differed in protein structure, according to the 3D-model prediction (Table 3). The template, 6xpd, and 6xpe were the best models for all Zn-CDF members (Cryo-EM structures of human ZnT8 in both outward- and inward-facing conformations). The 3h90.1 (structural basis for the autoregulation of the zinc transporter YiiP) was the best model for all Mn-CDF and Zn/Fe-CDF members, except HvMTP8.2, which was best modeled with 3J1Z (inward-facing conformation of the zinc transporter YiiP revealed by cryo-electron microscopy).

**Table 3:** Details of templates used for building 3D structure models of barley HvMTP proteins

Proteins name	Template	Sequence identity (%)	Coverage	GMQE	QMEANDisCo Global	Description
HvMTP1.1	6xpd	37.72	A46-413	0.50	0.63 ± 0.05	Zinc transporter 8
HvMTP1.2	6xpd	38.43	A46-421	0.49	0.62 ± 0.05	Zinc transporter 8
HvMTP1.3	6xpe	36.72	A64-408	0.44	0.59 ± 0.05	Zinc transporter 8
HvMTP5.1	6xpe	18.58	A94-362	0.43	0.50 ± 0.05	Zinc transporter 8
HvMTP6.1	3h90.1	25.98	A69-376	0.32	0.48 ± 0.05	Ferrous-iron efflux pump fieF
HvMTP7.1	3h90.1	19.71	A120-475	0.35	0.40 ± 0.05	Ferrous-iron efflux pump fieF
HvMTP8.1	3h90.1	19.57	A112-394	0.37	0.49 ± 0.05	Ferrous-iron efflux pump fieF
HvMTP8.2	3j1z	20	A97-388	0.39	0.46 ± 0.05	Cation efflux family protein
HvMTP9.1	3h90.1	17.45	A100-380	0.40	0.49 ± 0.05	Ferrous-iron efflux pump fieF
HvMTP11.1	3h90.1	19.27	A107-388	0.39	0.49 ± 0.05	Ferrous-iron efflux pump fieF
HvMTP11.2	3h90.1	19.12	A100-311	0.37	0.50 ± 0.05	Ferrous-iron efflux pump fieF
HvMTP12.1	6xpe	26.38	A380-796	0.18	0.48 ± 0.05	Zinc transporter 8

*Hv H. vulgare*, *MTP* metal-tolerance protein

To predict the ratio of alpha-helices, extended strands, beta-turns, and random coils in all HvMTP proteins, the SOPMA (self-optimized prediction method with alignment) method was used (Supplementary Table S5). The alpha-helix predominates among all secondary structure predictions of HvMTP proteins, ranging from 38.27 to 61.15%, followed by the random coil (24.25–36.14%), extended strand (8.52–19.36%), and beta-turn (2.06–8.58%). Additionally, protein glycosylation sites of HvMTPs were also determined in this study, as shown in Supplementary Table S6. The results indicated that six out of 12 HvMTP proteins have *N*-glycosylation sites. HvMTP1.1 and HvMTP1.2 had two and three *N*-glycosylation sites, respectively, while HvMTP1.3, HvMTP8.2, HvMTP9.1, and HvMTP12.1 consisted of one *N*-glycosylation site each. The derived homology models were verified using the Procheck Ramachandran plot analysis. The majority of the residues of HvMTPs were located in the preferred region of > 90.0% (Supplementary Figure S5). The homology model revealed that the overall structure of all the HvMTPs was very similar in terms of common strands and helices in the Rossmann folding type (Fig. 5).

### *Cis*-acting regulatory elements, microRNA target sites, and protein–protein interaction analysis of *HvMTP* genes

**Fig. 6:**
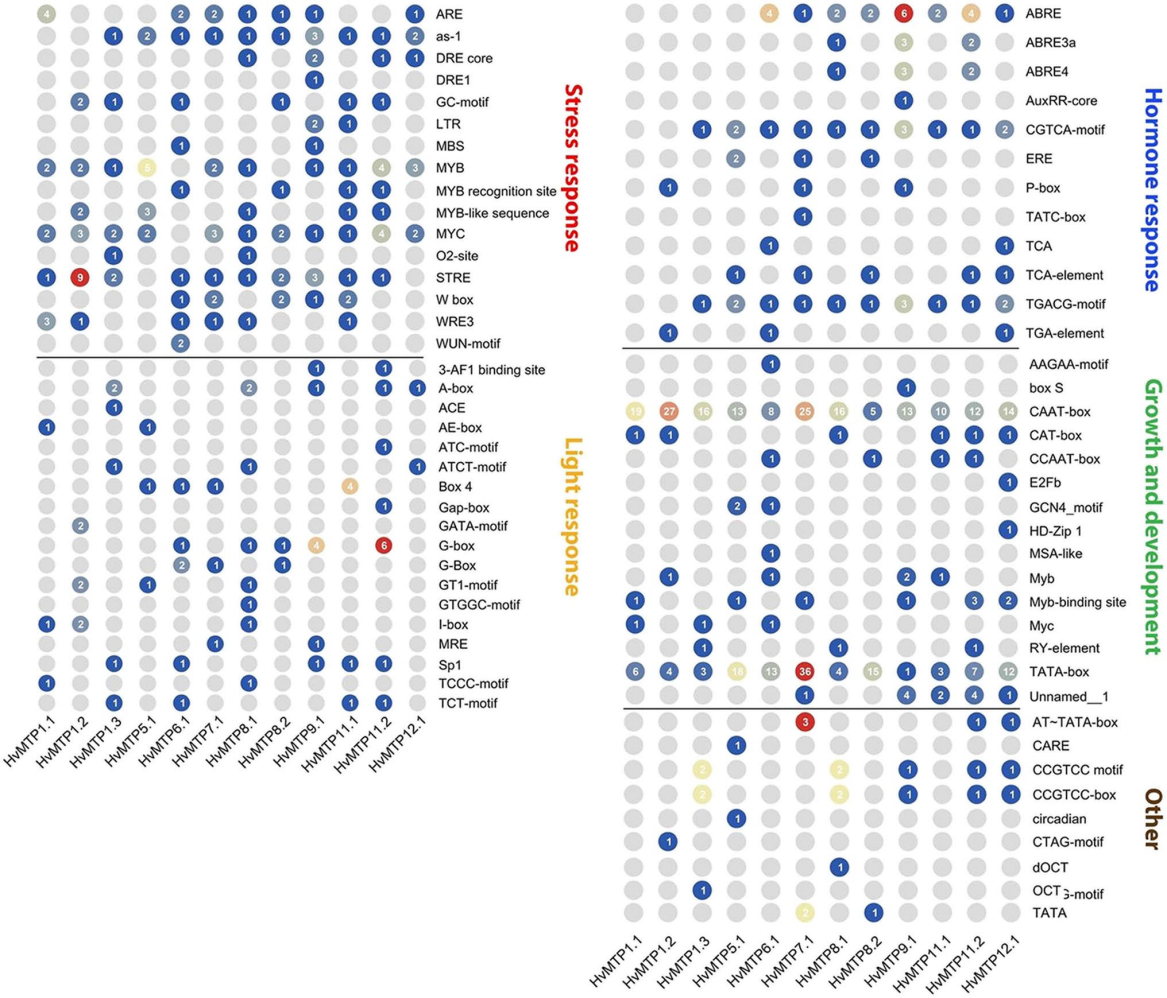
*Cis*-acting regulatory elements identified in the promoter regions of *HvMTP* genes. *Hv (H. vulgare)*; MTP (metal-tolerance protein)

To explore the probable regulatory mechanism of the expression of *HvMTP* genes, their CREs and microRNA target sites were predicted. A total of 666 CREs were identified in the promoter regions of *HvMTP* genes. These CREs were identified to be associated with hormone response (81), stress response (143), light response (65), growth and development (350), and other (27) (Fig. 6). The promoter of all *HvMTP* genes harbored CAAT and TATA-box (growth and development) with a total number of 178 and 122, respectively. Most *HvMTP* genes have hormone-responsive elements in the promoters except *HvMTP1.1*.

A total of three miRNAs target sites were identified in four *HvMTP* genes (Table 4) belonging to three groups. The UPE value varied from 19.284 (hvu-miR5051/HvMTP12.1) to 24.003 (hvu-miR6198/HvMTP11.1). The gene pairs of group 1, *HvMTP1.1* and *HvMTP1.2*, were predicted to be targets of hvu-miR6196 only and showed inhibition in the translation manner. *HvMTP12.1* and *HvMTP11.1* were potential target genes of hvu-miR5051 and hvu-miR6198, respectively, and inhibited by the corresponding miRNA in a cleavage manner.

**Table 4:** The potential miRNA target sites in *HvMTP* genes

miRNA	Target gene	Expectation	UPE	Target_start	Target_end	miRNA_aligned	Target_aligned	Inhibition
hvu-miR5051	*HvMTP12.1*	5	19.28	2368	2388	UUUGGCACCUUGAAACUGGGA	AUCCAGAUUGAAUGUGUCGAA	Cleavage
hvu-miR6196	*HvMTP1.1*	5	19.79	287	307	AGGACGAGGAGAUGGAGAGGA	CCUUUGCCAUAUCUUUGUUCU	Translation
hvu-miR6196	*HvMTP1.2*	5	22.91	287	307	AGGACGAGGAGAUGGAGAGGA	CAUUUGCCAUAUCUUUGUUCU	Translation
hvu-miR6198	*HvMTP11.1*	5	24.00	274	295	GCUCUGUCUUGGAUGGUCAUUC	CCUGGAAUGUCCAAGGAAGAGC	Cleavage

*Hv H. vulgare*, *MTP* metal-tolerance protein

**Fig. 7:**
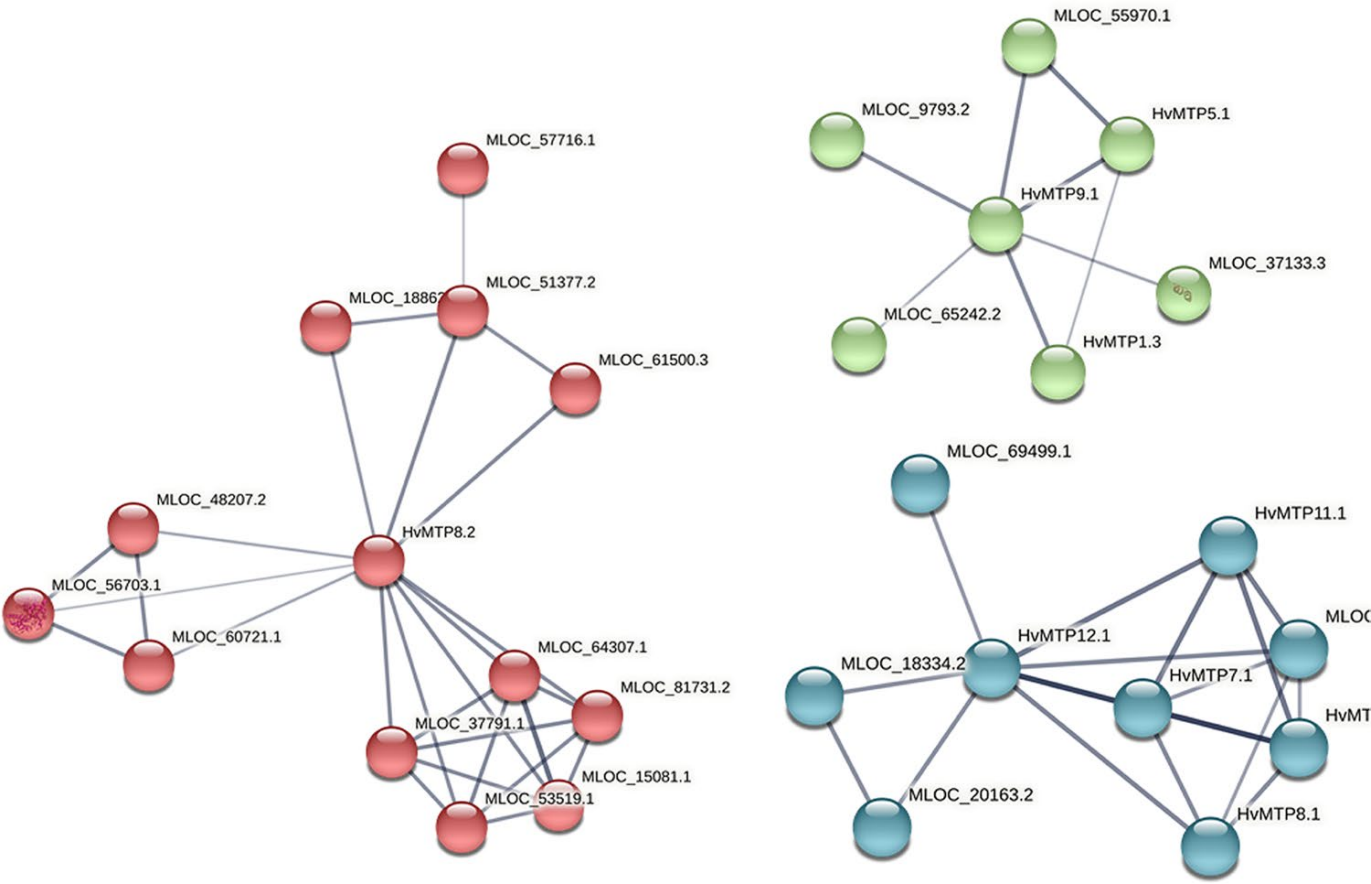
HvMTP protein clustering interaction network. Network nodes represent proteins; edges represent protein–protein associations. Nodes in red color represent cluster 1, green color represents cluster 2, and blue color represents cluster 3. *Hv (H. vulgare)*; MTP (metal-tolerance protein)

The protein–protein interactions analysis was carried out on the STRING webtool. Out of a total of 12 HvMTP proteins, 10 showed protein–protein interactions (Fig. 7), while HvMTP1.1 and HvMTP9.1 did not show any interaction. The protein interactions were further analyzed using *k*-means clustering and clustered in three clusters (Supplementary Table S7). Cluster 1 had only HvMTP8.2 which showed interactions with MLOC_15081.1 (ribonucleoside-diphosphate reductase), MLOC_18862.4 (Hma domain-containing protein), MLOC_37791.1 (Nusb domain-containing protein), MLOC_51377.2 (solute carrier family 40 protein), MLOC_56703.1 (epimerase domain-containing protein), MLOC_60721.1 (Fn3_like domain-containing protein), MLOC_64307.1 (guanylate kinase-like domain-containing protein), and MLOC_81731.2 (Ppr_long domain-containing protein). In cluster 2, there were three HvMTPs (HvMTP1.3, HvMTP5.1, and HvMTP9.1) that had interactions with MLOC_37133.3 (calcium-transporting ATPase), MLOC_65242.2 (LigB domain-containing protein), and MLOC_9793.2 (Wd_repeats_region domain-containing protein). Cluster 3 contained five HvMTPs (HvMTP11.1, HvMTP12.1, HvMTP6.1, HvMTP7.1, and HvMTP8.1) which showed interactions with MLOC_18334.2 (Mfs domain-containing protein), MLOC_20163.2 (Aa_trans domain-containing protein), and MLOC_69499.1 (bifunctional dihydrofolate reductase-thymidylate synthase).

### Expression analysis of *HvMTP* genes based on RNA-Seq data

**Fig. 8:**
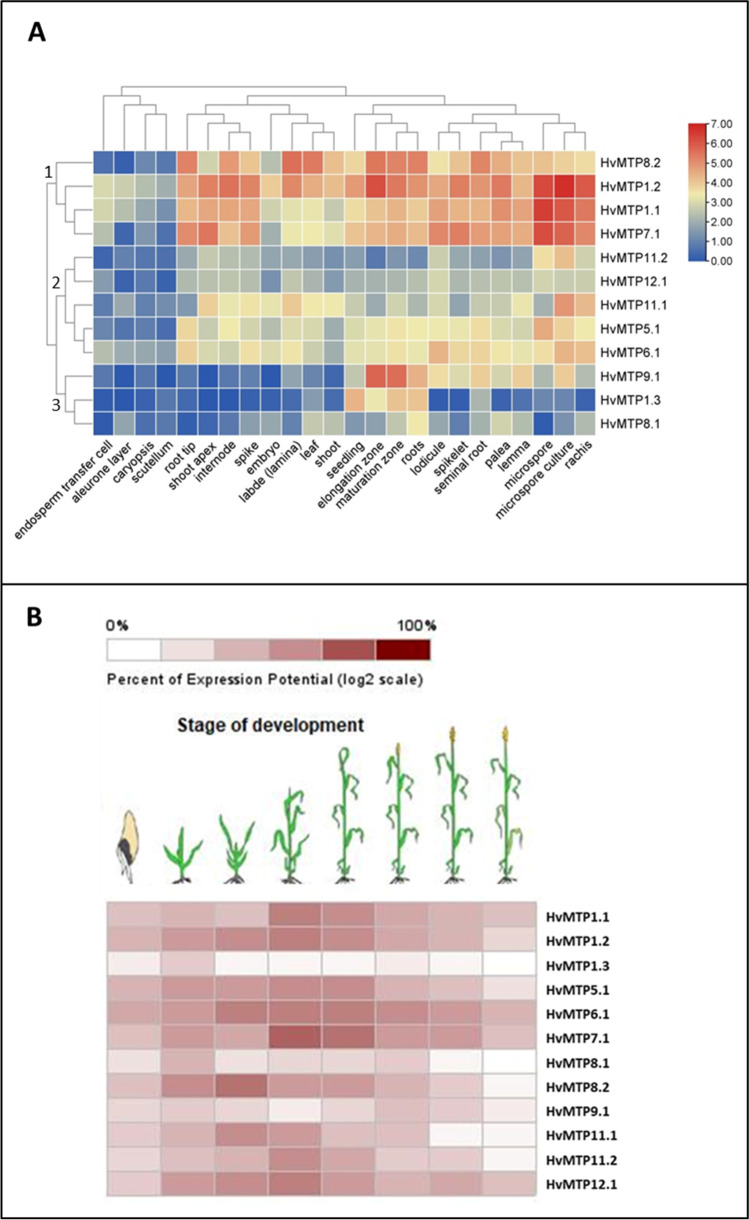
The expression profiles of *HvMTP* genes in different tissues (**A**) and developmental stages (**B**) of barley. The color scale shows the log2FC expression. *Hv (H. vulgare)*; MTP (metal-tolerance protein)

RNA-Seq data showed that most of the *HvMTP* genes are expressed in all barley tissues. To better understand the gene expression profiles, a hierarchical cluster analysis was carried out. As presented in Fig. 8A, 12 *HvMTP* genes were divided into three clusters: cluster 1, 2, and 3. All the genes were expressed at low levels in endosperm transfer cells, aleurone layer, caryopsis, and scutellum tissues. Cluster 1 includes 4 (*HvMTP8.2*, *HvMTP1.1*, *HvMTP1.2*, and *HvMTP7.1*) genes with high expression levels across all the tissues. Cluster 2 consists of five *HvMTP* genes including *HvMTP5.1*, *HvMTP6.1*, *HvMTP11.1/11.2*, and *HvMTP12.1*, with intermediate expression levels in approximately all tissues. *HvMTP5.1* and *HvMTP6.1* genes showed a similar expression pattern. Cluster 3 consists of three *HvMTP* genes including *HvMTP9.1*, *HvMTP1.3*, and *HvMTP8.1* with low expression levels in all the tissues; however, *HvMTP9.1* showed high expression in roots, elongation zone, and maturation zone.

Next, we analyzed gene expression of barley *HvMTP* genes at eight developmental stages, namely, germination, seedling, tillering, stem elongation, booting, flowering, milk, and dough stage (Fig. 8B). All the *HvMTP* genes were expressed at the germination stage, except *HvMTP1.3* (only at the seedling stage). All the genes were expressed during the seedling stage. Many genes such as *HvMTP1.1/1.2*, *HvMTP5.1*, *HvMTP6.1*, *HvMTP7.1*, and *HvMTP12.1* were expressed at all the developmental stages. These data indicate a correlative gene expression pattern of *HvMTP* genes, particularly during developmental stages.

**Fig. 9:**
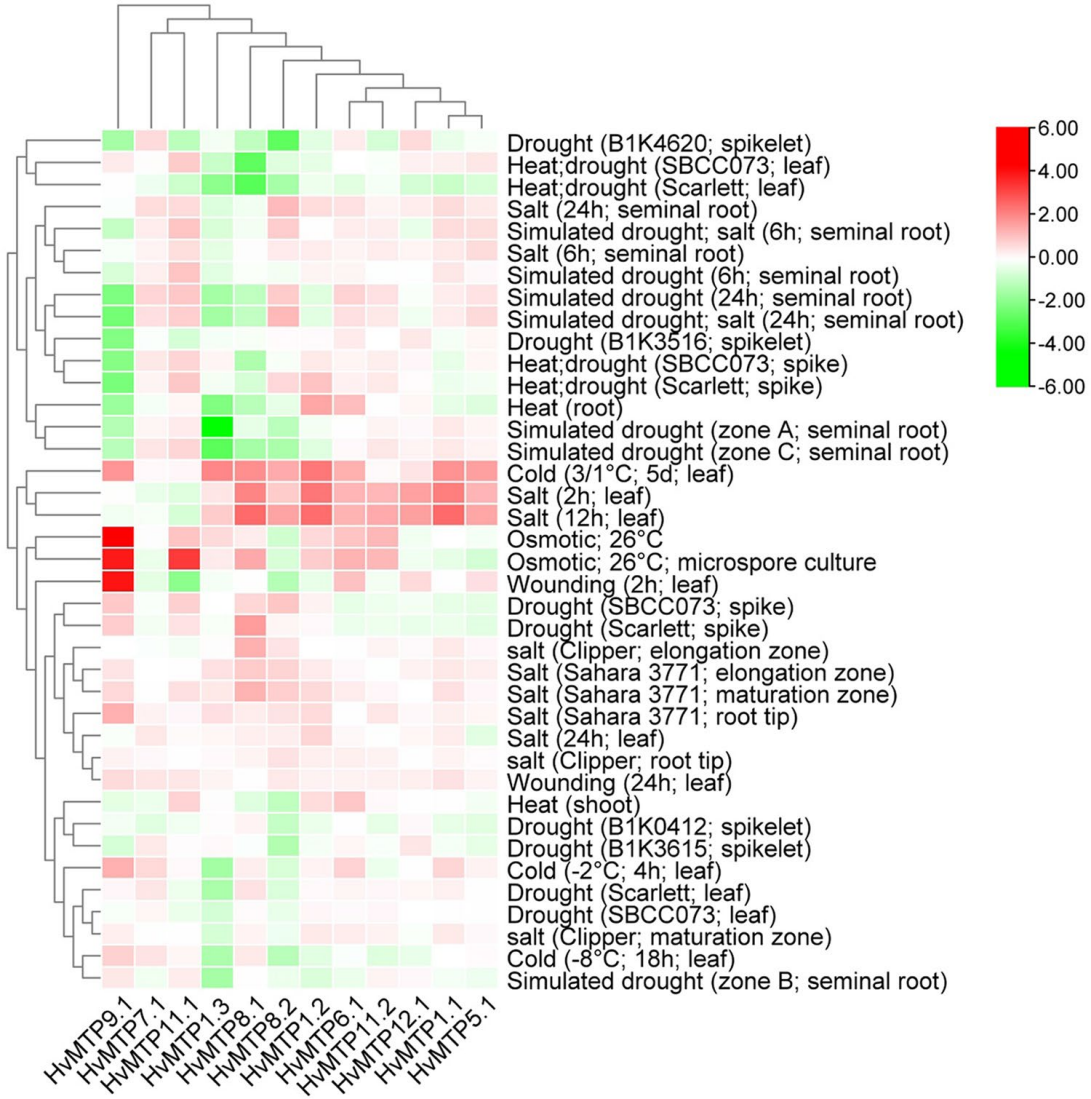
The expression profiles of *HvMTP* genes during various abiotic stress conditions in barley. The color scale shows the log2FC expression. *Hv (H. vulgare)*; MTP (metal-tolerance protein)

Expression of *HvMTP* genes was not found to significantly respond to various stress conditions (Fig. 9). *HvMTP9.1* was highly upregulated under osmotic stress conditions and wounding (2 h; leaf), while *HvMTP11.1* was highly upregulated under osmotic stress conditions. The *HvMTP1.3* gene was downregulated in simulated drought conditions in seminal roots. *HvMTP9.1* was downregulated in heat, drought, and simulated drought conditions. Most of the genes, except *HvMTP11.1/11.2*, *HvMTP12.1*, and *HvMTP7.1*, were slightly upregulated during cold and salt stress conditions. *HvMTP8.1/8.2* showed lower expression in heat/drought stress.

**Fig. 10:**
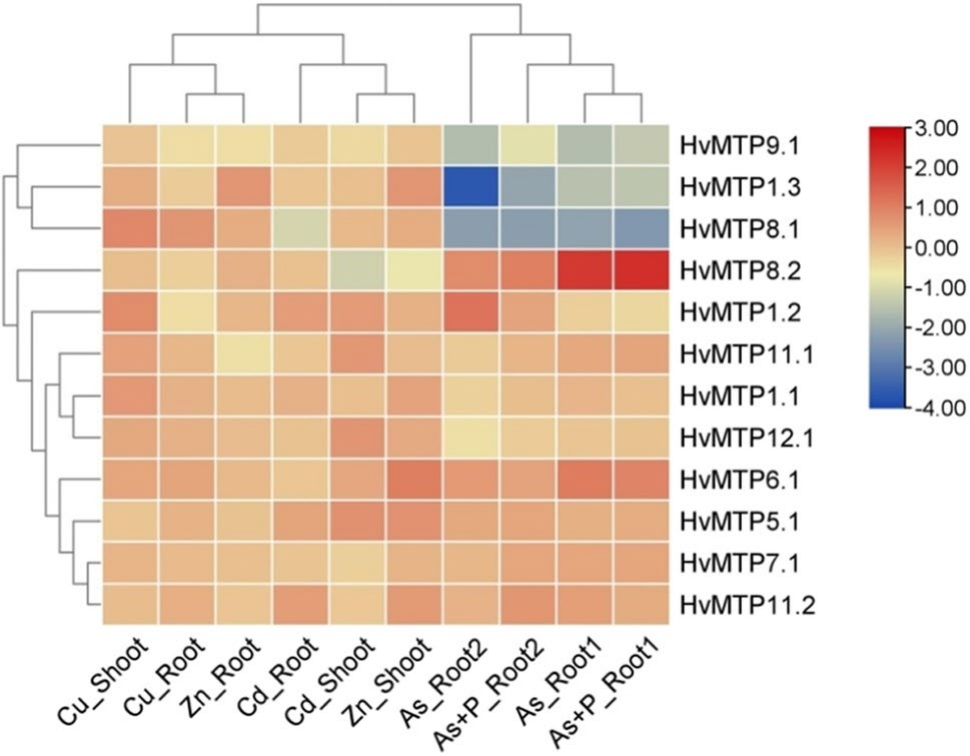
The expression profiles of *HvMTP* genes under heavy metal stress in barley. The color scale shows the log2FC expression. *Hv (H. vulgare)*; MTP (metal-tolerance protein)

We further analyzed the *HvMTP* genes expression during five heavy metal (arsenic, cadmium, phosphorus, zinc, and copper) stress in barley roots and shoots from the available RNA-Seq data. All the *HvMTP* genes showed differential expression patterns during the metal stress (Fig. 10). The genes *HvMTP9.1*, *HvMTP8.1*, and *HvMTP1.3* were downregulated during arsenic and phosphorus stress in roots. *HvMTP1.3* was found to be the lowest expressed under arsenic stress in roots. The expression of the rest of the genes, except *HvMTP8.1/8.2*, were upregulated during all metal stress conditions. *HvMTP8.2* was the highest expressed gene during arsenic and phosphorus stress in roots. *HvMTP8.1/8.2* genes were downregulated during cadmium stress in roots and shoots.

## Discussion

### Identification, phylogeny, and classification of *MTP* genes in barley

*MTP* genes encode membrane divalent cation transporters which participate in the mechanisms of transport and tolerance of various heavy metals and have become the subject of our genome-wide identification, characterization, and expression analysis in barley. In this study, 12 *HvMTP* genes were found in barley (Table 1) and classified into seven groups (1, 5, 6, 7, 8, 9, and 12), each of which belongs to one of the three major substrate-specific groups (Zn-CDFs, Zn/Fe-CDFs, and Mn-CDFs) (Fig. 1). Our findings were consistent with those of *Arabidopsis* (Montanini et al. 2007), rice (Gustin et al. 2011), turnip (Li et al. 2018), tobacco (Liu et al. 2019), and *Populus* (Gao et al. 2020), implying that *HvMTPs* may serve similar functions to their plant homologs. When compared to *Arabidopsis*, the barley genome included many *MTP* homologs for some *AtMTP*, however, *AtMTP2*, *AtMTP3*, and *AtMTP4* homologs were not present. This finding suggested that the *HvMTP* gene family may have undergone gene expansion and/or gene loss during the course of its evolutionary history, most likely as a result of polyploidization events. In addition, barley had more MTP family members than rice, which is probably due to the large size of the barley genome.

Analysis and prediction were done on the HvMTPs’ properties, such as CDS length, protein size, MW, pI, GRAVY, sub-cellular localization, and TMD number. The size and molecular weight of HvMTP12.1 (797 amino acids and 86.55 kDa) were much higher than those of other HvMTPs, suggesting that they might have different functions and undergone different evolutionary processes. Most of the HvMTP proteins possessed 4–6 TMDs (Supplementary Figure S1), which is in line with the findings in other plants (Kolaj-Robin et al. 2015; Lu and Fu 2007; Ricachenevsky et al. 2013), whereas HvMTP12.1 showed 11 TMDs that is similar with grapevine VvMTP12 (Shirazi et al. 2019) and citrus CitMTP12 (Fu et al. 2017).

### Gene structure, chromosomal localization, gene duplication, and syntenic gene analysis of *MTP* genes in barley

The exon–intron structure can provide extra information to support the gene family phylogenetic analyses (Zhang et al. 2012). In the current work, we discovered that HvMTP genes from the same evolutionary group have substantially comparable exon–intron architectures (Fig. 2A). Notably, four Zn-CDF genes in group 1 (*HvMTP1.1/1.2/1.3*) and 12 (*HvMTP12.1*) have one exon without an intron in the CDS, which are referred to as single exon genes (SEG) (Sakharkar et al. 2004). Given that SEG genes are prototypical of prokaryotes, their presence in multicellular eukaryotic genomes is intriguing (Sakharkar et al. 2004). Herein, *HvMTP1.1/1.2/1.3* having an intron in the UTR belong to uiSEGs (UTR intron-containing SEGs) while *HvMTP12.1* is IG (intronless genes).

Gene duplication has been identified as a significant source of new genes, contributing to the evolution of novel functionalities (Hittinger and Carroll 2007; Panchy et al. 2016). Gene duplication could be obtained from subgenomic duplication events other than whole-genome duplication (WGD), such as tandem and segmental duplication (Bailey et al. 2002; Zhang 2003). In this work, *HvMTP1.1/HvMTP1.2* was discovered as a segmental duplication event, which may result in the expansion of group 1 *HvMTPs*. The Ka/Ks value of the *HvMTP* gene pair was less than one, which supports that evolution of genes may have occurred from intensive purifying selection pressure by natural selection during the evolutionary process (Table 2). Furthermore, the gene pair (*HvMTP1.1/HvMTP1.2*) is newly duplicated, having diverged approximately 11.142 MYA after the genus *Hordeum* (12–13 MYA).

To further investigate the evolutionary relationships of *MTP* genes, we performed genome-to-genome synteny analysis between barley and four representative plant species. These included one dicot, *Arabidopsis*, and three monocots, *B. distachyon*, *O. sativa*, and *S. bicolor* (Fig. 3). Comparatively, the *MTP* genes in *Arabidopsis* showed the weakest (only 1) orthologous correlation. In general, compared to dicotyledons, monocotyledons showed higher synteny with the identified *HvMTP* members. Therefore, we speculated that the syntenic correlations between *MTP* members might be connected to the species’ evolutionary divergence. In particular, 8–10 *HvMTPs* were identified to be syntenic with *MTP* members across all the monocotyledons tested, indicating that these orthologous pairs are conserved and may have existed before the species divergence. The intersections of syntenic *MTP* members among different species may be useful for undertaking relevant *MTP* gene evolution studies.

In addition to the above, to more precisely understand the importance of *MTPs* in domestication of barley and its adaptation, we have assessed and outlined the variances in 20 barley pan-genome accessions between wild accession B1K-04–12 and other cultivated types, using Morex as an example. Strikingly, all studied varieties had the same number of MTPs, namely 12 (Supplementary Table S1). Taking into consideration the forces driving the selectivity of the proteins, we calculated the Ka/Ks ratio of homologous gene pairs of *HvMTP* in wild-type and cultivated materials. The values of Ka/Ks ratio (Ka/Ks < < 1) suggest that a large number of homologous genes underwent purification selection. Additionally, Ka/Ks values of some genes were equivalent to “NA” which suggests that no synonymous substitutions (Ks) are present in these genes (Supplementary Table S2). All of the above points that *HvMTPs* are very conserved genes in barley.

### Conserved motifs, domain architectures, and homology modeling of HvMTP proteins

Sequence analysis of HvMTP proteins showed the characteristic MTP family hallmarks (cation efflux and CDF), as previously described (Montanini et al. 2007). In the HvMTP proteins, among the identified conserved motifs, five motifs (2, 3, 4, 5, and 7) were identified as TMD or CTD of cation_efflux superfamilies (Supplementary Table S4). To assess metal selectivity, the histidine-rich loop in MTP was taken into account (Podar et al. 2012). We found that classic histidine-rich regions are present in groups 1 and 12 barley MTPs (Supplementary Fig. 2). The ability of HvMTPs to transport specific metal ions may be due to the variations in the length of the histidine-rich regions. The conservative sequences of HvMTPs (HxxxD and DxxxD) are diversely arranged in 3 clusters of these proteins (Supplementary Fig. 2–4). It has been shown previously that functional changes and selectivity to metal ions of different CDS groups may be associated with differences in amino acid sequences (Montanini et al. 2007).

What is more, in this study, we carried out the homology modeling of all the HvMTPs (Fig. 5), and the 3D structures of all the proteins showed the number of residues > 90% in the most favored region as per the Ramachandran plot analysis (Supplementary Fig. 5), which indicates the high accuracy of the structure prediction. Protein glycosylation is a critical component of protein structure that regulates a variety of biological processes (Corfield 2017). *N*-glycosylation sites for all HvMTP proteins were identified in this work, and *N*-glycosylation sites were found in 6 of the 12 HvMTPs (Supplementary Table S6) indicating their role in various cellular functions.

### *Cis*-acting regulatory elements, microRNA target sites, and protein–protein interaction analysis of *HvMTP* genes

We investigated the potential regulatory mechanism that controls the expression of *HvMTP* genes by looking into *cis*-acting regulatory elements (CREs) and miRNA target sites in the promoter regions and sequences coding *HvMTPs*, respectively. Here, we identified overall 666 putative CREs taking part in multiple biological processes (Fig. 6) and found a high number of CAAT-box and TATA-box elements in the promoter regions of *HvMTPs*. This is not surprising since those two types of sequences take part in regulating the frequency of expression and initiate the transcription (Laloum et al. 2013). Additionally, a large quantity of light-sensitive elements, phytohormone, and abiotic stress-responsive elements in the promoter regions imply that the *HvMTPs* expression might be controlled by a number of different elements which act through several pathways. Especially, *HvMTP7.1* seems to have the most numerous regulatory elements, which suggests that this gene may take part in many regulatory pathways. It has been proposed that MYB-binding sites (MBS) can be involved in metal tolerance by controlling the expression of *MTP1* in *Arabidopsis halleri* (Fasani et al. 2017). The presence of MBS in the promoters of *HvMTP6*.1 and *HvMTP9*.1 suggests that these genes may be controlled at MYB-binding sites.

In this study, three HvmiRNAs with four target sites were identified (Table 4). These miRNAs might be involved in both abiotic and biotic stress responses in barley. For instance, upregulation of hvu-miR6196 occurs during salt adaptation of the autopolyploid *Hordeum bulbosum* (Liu and Sun 2017) and during barley exposition to an excess of boron (Unver and Tombuloglu 2020). Differential expression of hvu-miR6198 was reported in Tibetan hulless barley during barley leaf stripe disease development after *Pyrenophora graminea* infection (Yao et al. 2021). Thus, exploring the role of miRNAs in *HvMTP* gene functions in response to various stresses would be of great interest.

The regulatory PPI network for the barley HvMTP proteins also indicated considerable interactive networks among the several other proteins (Fig. 7). This also supports the CREs and microRNA analysis that HvMTPs play various roles in plant development and stress tolerance. For example, Hma domain-containing protein, which showed interaction with HvMTP8.2, might be the protein associated with the heavy metal stress resistance of barley (Zhang et al. 2021).

### Expression analysis of *HvMTP* genes based on RNA-Seq data

In order to prepare foundations for determining the physiological functions of MTPs in barley, we analyzed the expression level of *HvMTPs* in organs in a time course, using publicly available transcriptomic data. The expression profiles present in tissues show that 50% of *HvMTP* genes are expressed at a low level in various localizations and during different developmental stages of plants. Among these genes, one (*HvMTP8.1*) was rarely or not at all expressed in any tissues (Fig. 8). The very low expression level of this gene may be advantageous for keeping its biological functions and avoiding the loss of the gene during evolution (Liu et al. 2019; Qian et al. 2010). Out of the remaining 12 *HvMTP* genes, three had very high expression levels in almost all tissues, namely *HvMTP8*.2, *HvMTP1.1/1.2*, and *HvMTP7*.1. These genes may be important players in controlling the development and growth processes in plants by facilitating the transport of divalent metals. Additionally, three more genes, *HvMTP1*.2, *HvMTP8.2*, and *HvMTP9*.*1*, are highly expressed in the roots elongation zone and maturation zone. Although most of the HvMTP proteins are predicted to be localized in vacuolar membranes, the studies on the localization of MTP9 in rice showed that this protein is localized in plasma membrane in the proximal side of the exodermis and endodermis cells and it plays a role in radial transport of Mn out of the cells and toward the stele for xylem loading and translocation to the shoots (Chang et al. 2020; Ueno et al. 2015). However, we believe that altering the expression levels of *HvMTPs* in roots may change the root-to-shoot transport of divalent metals. High expression levels of *HvMTP1*.2 and *HvMTP8*.2 in leaves indicate that these genes may take part in metal homeostasis in these organs. What is interesting, *HvMTP1.1/1.2* and *HvMTP7*.1 were highly expressed in the endosperm and aleurone layer. This is in agreement with results obtained for wheat, since the *TaMTP1s*, *TaMTP2s*, and *TaMTP7A* are expressed at high levels in wheat endosperm. Most of the Fe, Mn, and Zn are found in the aleurone layer of seeds and the embryo; while the endosperm, which is the largest part of the seed, is poor in these ions (Borg et al. 2009; Mazzolini et al. 1985; Regvar et al. 2011). Taking into account that HvMTPs are typically placed in the tonoplast, it seems that they take part in building the reserves in cell vacuoles in the aleurone layer of barley grains. Particularly, because the nearest orthologs of HvMTP1s were characterized as a Zn transporter associated with tonoplast in *Arabidopsis* and rice, *HvMTP1s* may be responsible for higher Zn levels in the vacuoles of the aleurone tissue. All of the abovementioned suggest that temporal and spatial regulation of *MTPs* genes followed by MTPs abundance play a crucial role in maintaining nutrient homeostasis throughout the life cycle of barley. Another interesting result of our study is the prediction of sub-cellular localization of HvMTP8.1 and HvMTP8.2 to tonoplast. However, Pedas et al. (2014) demonstrated that these proteins are associated with Golgi apparatus. Although the study of Pedas et al. (2014) focuses on barley MTPs, the sub-cellular localization experiments were done in *Allium cepa.* Hence, to dispel any doubts, additional “wet-lab” experiments on the localization of these proteins should be performed, e.g., western blot analysis using appropriate fractions of cell membranes.

Using publicly available RNA-Seq datasets, we also analyzed the expression profiles of identified *HvMTPs* in response to different types of stresses, heavy metal stress being one of them. Generally, all of the genes are being upregulated in plants exposed to short-term and prolonged salt stress as well as in leaves exposed to cold (Fig. 9). Upregulation of barley *MTPs* under salinity is expected since metal toxicity induces similar responses as the salt stress since both can disturb the ionic balance and in consequence limit crop production (Chinnusamy et al. 2005; Li et al. 2010) Interestingly such an effect, of increased expression, is not observed with drought and/or heat stress. This can be especially seen for *HvMTP1.3*, *HvMTP8.1*, and *HvMTP8.2*, which are downregulated in these stresses. Interestingly, in response to osmotic stress, the most upregulated *MTP* is *MTP9.* According to Migocka et al. (Migocka et al. 2015) and Shirazi et al. (2019), the MTP9 protein may take part in an increase of Mn and Cd accumulation in shoots, making it useful for phytoremediation of soils contaminated with these two heavy metals. These results indicate that the role of the *MTPs* products in response to various stresses is multilayered and varies in different species, and the investigation on protein level and protein interactions would be of great interest for future studies to fully understand their complexity.

Furthermore, literature data indicate that *MTPs* play a key role in increasing plant tolerance against heavy metals (Gao et al. 2020; Montanini et al. 2007) as the metal transporters primarily transport Zn^2+^, but they are also able to conduct other divalent cations like Ni^+2^, Co^2+^, Cd^2+^, Fe^2+^, and Mn^2+^ (Ricachenevsky et al. 2013). Hence, a search for the expression behavior of *MTP* genes was conducted under heavy metal stress. The expression of all *MTP* genes was induced by at least three metals both in roots and shoot, even though some were not putative substrates for the transporters. For example, an increased concentration of Cu, which is theoretically not a substrate for MTP proteins, increases the expression of all identified *HvMTPs* (Fig. 10). Cd is a non-essential element for plants, and they do not have distinct mechanisms for the uptake of cadmium ions, but they can be absorbed and transported utilizing some of the carriers used for the uptake of other metals necessary for plant development (Li et al. 2017). Similar upregulation of *MTPs* in response to Cd was also found in *Brassica rapa* and *Citrus sinensis* (Fu et al. 2017; Li et al. 2018). This indicates the potential role of barley *MTPs* genes in cadmium detoxification. The upregulation of certain isoforms may be not only due to response to specific ion presence but also due to the formation of complexes with other isoforms. As it was shown in other plants, for example, increased expression of AtMTP12 is not caused by treatment with zinc, but due to the interaction of its product with AtMTP5, which leads to Zn^2+^ transport (El-Sappah et al. 2021; Fujiwara et al. 2015; Liu et al. 2019), it would be desirable to study the activity of HvMTP proteins in response to heavy metals and identify the potential MTPs involved in the formation of heterocomplexes in barley to unravel their affinity to different ions in future studies.

Overall, this study provides significant fundamental information on the possible functions of *HvMTP* genes, which will benefit efforts toward functional characterization for metal tolerance, bioremediation, or important topics such as crop biofortification. Food is the only source of metal micronutrients for humans, and plants are the primary source of these metals in developing countries. Mineral content and homeostasis maintenance in plant edible tissues are thus critical for human nutrition. Grain biofortification has been proposed as a promising strategy for improving human nutrition, and in this direction, the investigation and modification of expression of genes encoding metal transporters, either alone or in combination with other genes, might be a useful approach. The present study establishes the framework for additional experimental exploration of *MTP* genes and provides important gene resources for the genetic improvement of barley crop.

## Supplementary Information

Below is the link to the electronic supplementary material.

Supplementary file1 (DOCX 562 KB)

Supplementary file2 (DOCX 2449 KB)

## Data Availability

The data that support the findings of this study are available at Ensembl Plants database (https://plants.ensembl.org/index.html) or from the corresponding author upon reasonable request.
