# Supplementary material for: Metal tolerance gene family in barley: an in silico comprehensive analysis
Source: J Appl Genet. 2022 Dec 31;64(2):197–215. doi: 10.1007/s13353-022-00744-6 (PMC10076399; doi:10.1007/s13353-022-00744-6)
Supplement: Supplementary file 1 — Supplementary file1 (DOCX 562 KB) [file 13353_2022_744_MOESM1_ESM.docx]

Supplementary Table S1. Number of MTP genes in each accession of the barley pan-genome.

| **Accessions** | **Number of HvMTPs** |
| --- | --- |
| Akashinriki | 12 |
| B1K-04-12 | 12 |
| Barke | 12 |
| Golden Promise | 12 |
| Hockett | 12 |
| HOR3081 | 12 |
| HOR3365 | 12 |
| HOR7552 | 12 |
| HOR8148 | 12 |
| HOR9043 | 12 |
| HOR10350 | 12 |
| HOR13821 | 12 |
| HOR13942 | 12 |
| HOR21599 | 12 |
| Igri | 12 |
| Morex | 12 |
| OUN333 | 12 |
| RGT_Planet | 12 |
| ZDM01467 | 12 |
| ZDM02064 | 12 |

Supplementary Table S2. Ka/Ks values of MTPs in Morex and B1K-04-12 accessions of barley

| **Seq_1 (Morex)** | **Seq_2 (B1K-04-12** | **Ka** | **Ks** | **Ka_Ks** |
| --- | --- | --- | --- | --- |
| HORVU.MOREX.r3.1HG0073140.1 | Horvu_FT11_1H01G448300.1 | 0.086401 | 0.805502 | 0.107264 |
| HORVU.MOREX.r3.1HG0016110.1 | Horvu_FT11_1H01G100500.1 | 0 | 0 | NA |
| HORVU.MOREX.r3.1HG0073140.1 | Horvu_FT11_3H01G530000.1 | 0.001111 | 0.020508 | 0.054194 |
| HORVU.MOREX.r3.2HG0158870.1 | Horvu_FT11_2H01G379500.1 | 0 | 0 | NA |
| HORVU.MOREX.r3.2HG0174930.1 | Horvu_FT11_2H01G476500.1 | 0.001042 | 0.007203 | 0.144717 |
| HORVU.MOREX.r3.2HG0119060.1 | Horvu_FT11_2H01G159000.1 | 0 | 0.003384 | 0 |
| HORVU.MOREX.r3.3HG0303160.1 | Horvu_FT11_1H01G448300.1 | 0.00137 | 0.004258 | 0.321762 |
| HORVU.MOREX.r3.3HG0222000.2 | Horvu_FT11_3H01G025500.1 | 0 | 0.010817 | 0 |
| HORVU.MOREX.r3.3HG0303160.1 | Horvu_FT11_3H01G530000.1 | 0.100041 | 0.726824 | 0.137641 |
| HORVU.MOREX.r3.4HG0393950.1 | Horvu_FT11_4H01G359200.1 | 0.196299 | NA | NA |
| HORVU.MOREX.r3.4HG0409880.1 | Horvu_FT11_4H01G471900.1 | 0.001064 | 0.014261 | 0.074586 |
| HORVU.MOREX.r3.4HG0376610.1 | Horvu_FT11_4H01G254300.1 | 0 | 0 | NA |
| HORVU.MOREX.r3.6HG0617950.1 | Horvu_FT11_6H01G475900.1 | 0.195912 | NA | NA |
| HORVU.MOREX.r3.6HG0634070.1 | Horvu_FT11_6H01G587600.1 | 0.006286 | 0.043928 | 0.143098 |

Supplementary Table 3: Syntenic relationship between barley and other plants, *A. thaliana*, *O. sativa, B. distachyon,* and *S. bicolor* MTPs.

| **Barley chromosome number** | **Barley *MTP*** | **Chromosome number in other species** | **MTP in other species** | **Species name** |
| --- | --- | --- | --- | --- |
| chr3H | HORVU.MOREX.r3.3HG0303160.1.V3 | Chr2 | AT2G39450.1 | *A. thaliana* |
| chr1H | HORVU.MOREX.r3.1HG0073140.1.V3 | Chr1 | LOC_Os01g62070.1 | *O. sativa* |
| chr1H | HORVU.MOREX.r3.1HG0016110.1.V3 | Chr5 | LOC_Os05g03780.1 | *O. sativa* |
| chr1H | HORVU.MOREX.r3.1HG0073140.1.V3 | Chr5 | LOC_Os05g38670.1 | *O. sativa* |
| chr4H | HORVU.MOREX.r3.4HG0393950.1.V3 | Chr3 | LOC_Os03g12530.1 | *O. sativa* |
| chr4H | HORVU.MOREX.r3.4HG0376610.1.V3 | Chr3 | LOC_Os03g22550.1 | *O. sativa* |
| chr3H | HORVU.MOREX.r3.3HG0303160.1.V3 | Chr1 | LOC_Os01g62070.1 | *O. sativa* |
| chr3H | HORVU.MOREX.r3.3HG0222000.2.V3 | Chr1 | LOC_Os01g03914.1 | *O. sativa* |
| chr3H | HORVU.MOREX.r3.3HG0303160.1.V3 | Chr5 | LOC_Os05g38670.1 | *O. sativa* |
| chr6H | HORVU.MOREX.r3.6HG0634070.1.V3 | Chr2 | LOC_Os02g58580.1 | *O. sativa* |
| chr6H | HORVU.MOREX.r3.6HG0617950.1.V3 | Chr2 | LOC_Os02g53490.1 | *O. sativa* |
| chr1H | HORVU.MOREX.r3.1HG0073140.1.V3 | 3 | transcript:EES03850 | *S. bicolor* |
| chr1H | HORVU.MOREX.r3.1HG0016110.1.V3 | 9 | transcript:OQU77333 | *S. bicolor* |
| chr4H | HORVU.MOREX.r3.4HG0393950.1.V3 | 1 | transcript:EER92593 | *S. bicolor* |
| chr4H | HORVU.MOREX.r3.4HG0376610.1.V3 | 1 | transcript:KXG39395 | *S. bicolor* |
| chr3H | HORVU.MOREX.r3.3HG0222000.2.V3 | 3 | transcript:KXG31976 | *S. bicolor* |
| chr3H | HORVU.MOREX.r3.3HG0303160.1.V3 | 3 | transcript:EES03850 | *S. bicolor* |
| chr6H | HORVU.MOREX.r3.6HG0634070.1.V3 | 4 | transcript:EES06081 | *S. bicolor* |
| chr6H | HORVU.MOREX.r3.6HG0617950.1.V3 | 4 | transcript:KXG31199 | *S. bicolor* |
| chr1H | HORVU.MOREX.r3.1HG0073140.1.V3 | 2 | transcript:KQK05871 | *B. distachyon* |
| chr1H | HORVU.MOREX.r3.1HG0073140.1.V3 | 2 | transcript:KQK10488 | *B. distachyon* |
| chr1H | HORVU.MOREX.r3.1HG0016110.1.V3 | 2 | transcript:KQK07905 | *B. distachyon* |
| chr4H | HORVU.MOREX.r3.4HG0393950.1.V3 | 1 | transcript:KQK22713 | *B. distachyon* |
| chr4H | HORVU.MOREX.r3.4HG0376610.1.V3 | 1 | transcript:KQK21720 | *B. distachyon* |
| chr3H | HORVU.MOREX.r3.3HG0303160.1.V3 | 2 | transcript:KQK10488 | *B. distachyon* |
| chr3H | HORVU.MOREX.r3.3HG0303160.1.V3 | 2 | transcript:KQK05871 | *B. distachyon* |
| chr3H | HORVU.MOREX.r3.3HG0222000.2.V3 | 2 | transcript:KQK02527 | *B. distachyon* |
| chr6H | HORVU.MOREX.r3.6HG0617950.1.V3 | 3 | transcript:KQK01662 | *B. distachyon* |
| chr6H | HORVU.MOREX.r3.6HG0634070.1.V3 | 3 | transcript:KQK02181 | *B. distachyon* |

Supplementary Table S4: Analysis of 15 conserved motifs identified in HvMTP proteins.

| **Motif** | **Annotation** |
| --- | --- |
| 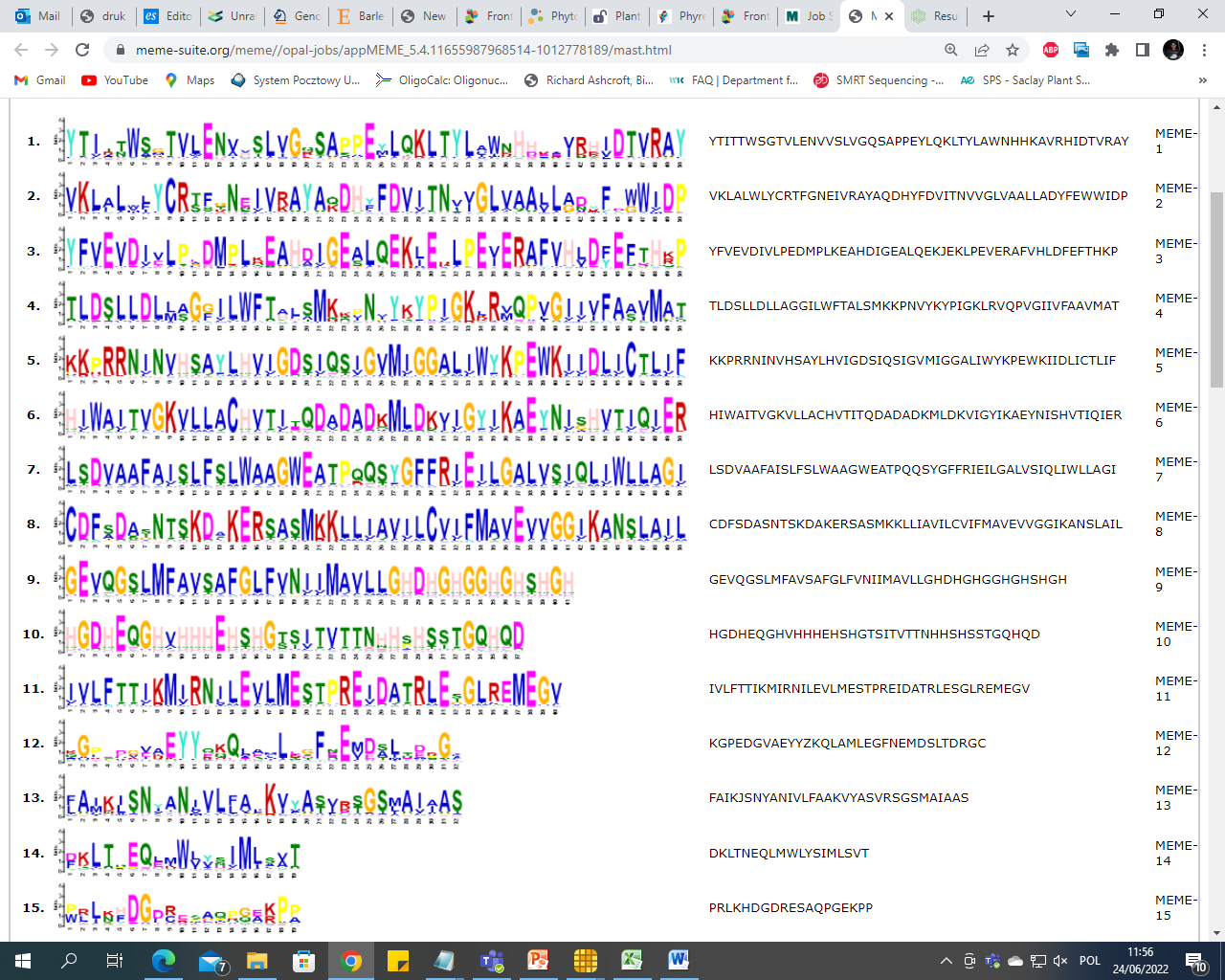 | ND |
|  | Cation_efflux_TMD_sf |
|  | Cation_efflux_CTD_sf |
|  | Cation_efflux_TMD_sf |
|  | Cation_efflux_TMD_sf |
|  | ND |
|  | Cation_efflux_TMD_sf |
|  | ND |
|  | ND |
|  | ND |
|  | ND |
|  | ND |
|  | ND |
|  | ND |
|  | ND |

Supplementary Table S5: Details of secondary structure prediction of HvMTP proteins.

| **HvMTP1.1** |
| --- |
| Alpha helix (Hh) : 180 is 43.58% |
| 310 helix (Gg) : 0 is 0.00% |
| Pi helix (Ii) : 0 is 0.00% |
| Beta bridge (Bb) : 0 is 0.00% |
| Extended strand (Ee) : 83 is 20.10% |
| Beta turn (Tt) : 34 is 8.23% |
| Bend region (Ss) : 0 is 0.00% |
| Random coil (Cc) : 116 is 28.09% |
| Ambiguous states (?) : 0 is 0.00% |
| Other states : 0 is 0.00% |
|  |
| **HvMTP1.2** |
| Alpha helix (Hh) : 164 is 38.95% |
| 310 helix (Gg) : 0 is 0.00% |
| Pi helix (Ii) : 0 is 0.00% |
| Beta bridge (Bb) : 0 is 0.00% |
| Extended strand (Ee) : 88 is 20.90% |
| Beta turn (Tt) : 31 is 7.36% |
| Bend region (Ss) : 0 is 0.00% |
| Random coil (Cc) : 138 is 32.78% |
| Ambiguous states (?) : 0 is 0.00% |
| Other states : 0 is 0.00% |
|  |
| **HvMTP1.3** |
| Alpha helix (Hh) : 171 is 41.91% |
| 310 helix (Gg) : 0 is 0.00% |
| Pi helix (Ii) : 0 is 0.00% |
| Beta bridge (Bb) : 0 is 0.00% |
| Extended strand (Ee) : 79 is 19.36% |
| Beta turn (Tt) : 35 is 8.58% |
| Bend region (Ss) : 0 is 0.00% |
| Random coil (Cc) : 123 is 30.15% |
| Ambiguous states (?) : 0 is 0.00% |
| Other states : 0 is 0.00% |
|  |
| **HvMTP5.1** |
| Alpha helix (Hh) : 179 is 49.45% |
| 310 helix (Gg) : 0 is 0.00% |
| Pi helix (Ii) : 0 is 0.00% |
| Beta bridge (Bb) : 0 is 0.00% |
| Extended strand (Ee) : 52 is 14.36% |
| Beta turn (Tt) : 12 is 3.31% |
| Bend region (Ss) : 0 is 0.00% |
| Random coil (Cc) : 119 is 32.87% |
| Ambiguous states (?) : 0 is 0.00% |
| Other states : 0 is 0.00% |
|  |
| **HvMTP6.1** |
| Alpha helix (Hh) : 264 is 52.59% |
| 310 helix (Gg) : 0 is 0.00% |
| Pi helix (Ii) : 0 is 0.00% |
| Beta bridge (Bb) : 0 is 0.00% |
| Extended strand (Ee) : 46 is 9.16% |
| Beta turn (Tt) : 19 is 3.78% |
| Bend region (Ss) : 0 is 0.00% |
| Random coil (Cc) : 173 is 34.46% |
| Ambiguous states (?) : 0 is 0.00% |
| Other states : 0 is 0.00% |
|  |
| **HvMTP7.1** |
| Alpha helix (Hh) : 243 is 52.71% |
| 310 helix (Gg) : 0 is 0.00% |
| Pi helix (Ii) : 0 is 0.00% |
| Beta bridge (Bb) : 0 is 0.00% |
| Extended strand (Ee) : 50 is 10.85% |
| Beta turn (Tt) : 26 is 5.64% |
| Bend region (Ss) : 0 is 0.00% |
| Random coil (Cc) : 142 is 30.80% |
| Ambiguous states (?) : 0 is 0.00% |
| Other states : 0 is 0.00% |
|  |
| **HvMTP8.1** |
| Alpha helix (Hh) : 231 is 56.34% |
| 310 helix (Gg) : 0 is 0.00% |
| Pi helix (Ii) : 0 is 0.00% |
| Beta bridge (Bb) : 0 is 0.00% |
| Extended strand (Ee) : 40 is 9.76% |
| Beta turn (Tt) : 15 is 3.66% |
| Bend region (Ss) : 0 is 0.00% |
| Random coil (Cc) : 124 is 30.24% |
| Ambiguous states (?) : 0 is 0.00% |
| Other states : 0 is 0.00% |
|  |
| **HvMTP8.2** |
| Alpha helix (Hh) : 243 is 60.75% |
| 310 helix (Gg) : 0 is 0.00% |
| Pi helix (Ii) : 0 is 0.00% |
| Beta bridge (Bb) : 0 is 0.00% |
| Extended strand (Ee) : 41 is 10.25% |
| Beta turn (Tt) : 19 is 4.75% |
| Bend region (Ss) : 0 is 0.00% |
| Random coil (Cc) : 97 is 24.25% |
| Ambiguous states (?) : 0 is 0.00% |
| Other states : 0 is 0.00% |
|  |
| **HvMTP9.1** |
| Alpha helix (Hh) : 220 is 56.56% |
| 310 helix (Gg) : 0 is 0.00% |
| Pi helix (Ii) : 0 is 0.00% |
| Beta bridge (Bb) : 0 is 0.00% |
| Extended strand (Ee) : 45 is 11.57% |
| Beta turn (Tt) : 8 is 2.06% |
| Bend region (Ss) : 0 is 0.00% |
| Random coil (Cc) : 116 is 29.82% |
| Ambiguous states (?) : 0 is 0.00% |
| Other states : 0 is 0.00% |
|  |
| **HvMTP11.1** |
| Alpha helix (Hh) : 244 is 61.15% |
| 310 helix (Gg) : 0 is 0.00% |
| Pi helix (Ii) : 0 is 0.00% |
| Beta bridge (Bb) : 0 is 0.00% |
| Extended strand (Ee) : 34 is 8.52% |
| Beta turn (Tt) : 16 is 4.01% |
| Bend region (Ss) : 0 is 0.00% |
| Random coil (Cc) : 105 is 26.32% |
| Ambiguous states (?) : 0 is 0.00% |
| Other states : 0 is 0.00% |
|  |
| **HvMTP11.2** |
| Alpha helix (Hh) : 166 is 51.55% |
| 310 helix (Gg) : 0 is 0.00% |
| Pi helix (Ii) : 0 is 0.00% |
| Beta bridge (Bb) : 0 is 0.00% |
| Extended strand (Ee) : 42 is 13.04% |
| Beta turn (Tt) : 21 is 6.52% |
| Bend region (Ss) : 0 is 0.00% |
| Random coil (Cc) : 93 is 28.88% |
| Ambiguous states (?) : 0 is 0.00% |
| Other states : 0 is 0.00% |
|  |
| **HvMTP12.1** |
| Alpha helix (Hh) : 305 is 38.27% |
| 310 helix (Gg) : 0 is 0.00% |
| Pi helix (Ii) : 0 is 0.00% |
| Beta bridge (Bb) : 0 is 0.00% |
| Extended strand (Ee) : 146 is 18.32% |
| Beta turn (Tt) : 58 is 7.28% |
| Bend region (Ss) : 0 is 0.00% |
| Random coil (Cc) : 288 is 36.14% |
| Ambiguous states (?) : 0 is 0.00% |
| Other states : 0 is 0.00% |

Supplementary Table S6. Details of N-glycosylation sites in HvMTP proteins

**SeqName Position Potential Jury N-Glyc**

**agreement result**

**----------------------------------------------------------------------**

**HvMTP1.1 5 NSSQ 0.6964 (9/9) ++**

**HvMTP1.1 42 NTSK 0.6700 (9/9) ++**

**HvMTP1.1 151 NESG 0.5259 (5/9) +**

**HvMTP1.1 402 NISH 0.5190 (6/9) +**

**HvMTP1.2 5 NSSP 0.2126 (8/9) --**

**HvMTP1.2 42 NTSK 0.6780 (9/9) ++**

**HvMTP1.2 151 NESG 0.6143 (9/9) ++**

**HvMTP1.2 239 NHSH 0.2986 (9/9) ---**

**HvMTP1.2 410 NISH 0.5188 (6/9) +**

**HvMTP1.3 3 NHTS 0.7418 (9/9) ++**

**HvMTP1.3 37 NTSK 0.6120 (8/9) +**

**HvMTP1.3 146 NGSG 0.6519 (8/9) +**

**HvMTP8.2 201 NVTP 0.1943 (9/9) ---**

**HvMTP8.2 287 NWSG 0.5606 (6/9) +**

**HvMTP9.1 104 NLSN 0.7543 (9/9) +++**

**HvMTP12.1 749 NLTN 0.6734 (9/9) ++**

Supplementary Table S7. The protein-protein interactions and kmeans clustering ofs HvMTP proteins.

| **clustering method** | **cluster number** | **cluster color** | **gene count** | **protein name** | **protein identifier** | **protein description** |
| --- | --- | --- | --- | --- | --- | --- |
| kmeans | 1 | Red | 7 | A0A287QRP4 | 112509.A0A287QRP4 | Uncharacterized protein |
| kmeans | 1 | Red | 7 | A0A287S0F4 | 112509.A0A287S0F4 | Uncharacterized protein |
| kmeans | 1 | Red | 7 | A0A287X8C3 | 112509.A0A287X8C3 | Solute carrier family 40 protein; May be involved in iron transport and iron homeostasis. ; Belongs to the ferroportin (FP) (TC 2.A.100) family. SLC40A subfamily. |
| kmeans | 1 | Red | 7 | A0A287XPG0 | 112509.A0A287XPG0 | Uncharacterized protein |
| kmeans | 1 | Red | 7 | F2CX26 | 112509.F2CX26 | Predicted protein |
| kmeans | 1 | Red | 7 | F2DJI5 | 112509.F2DJI5 | Predicted protein |
| kmeans | 1 | Red | 7 | HvMTP1.2 | 112509.A0A287EQC3 | Uncharacterized protein |
| kmeans | 2 | Green | 18 | A0A287EXG1 | 112509.A0A287EXG1 | HECT domain-containing protein |
| kmeans | 2 | Green | 18 | A0A287HGH3 | 112509.A0A287HGH3 | Uncharacterized protein |
| kmeans | 2 | Green | 18 | A0A287I1X5 | 112509.A0A287I1X5 | Uncharacterized protein |
| kmeans | 2 | Green | 18 | A0A287NW16 | 112509.A0A287NW16 | Rubredoxin-like domain-containing protein |
| kmeans | 2 | Green | 18 | A0A287QN38 | 112509.A0A287QN38 | Tubby-like F-box protein; Belongs to the TUB family. |
| kmeans | 2 | Green | 18 | A0A287QWV6 | 112509.A0A287QWV6 | Tubby-like F-box protein; Belongs to the TUB family. |
| kmeans | 2 | Green | 18 | A0A287TQZ2 | 112509.A0A287TQZ2 | Tub domain-containing protein; Belongs to the TUB family. |
| kmeans | 2 | Green | 18 | F2E053 | 112509.F2E053 | Tubby-like F-box protein; Belongs to the TUB family. |
| kmeans | 2 | Green | 18 | HvMTP1.3 | 112509.A0A287PUY8 | Uncharacterized protein |
| kmeans | 2 | Green | 18 | HvMTP11.1 | 112509.A0A287M3L2 | ZT_dimer domain-containing protein |
| kmeans | 2 | Green | 18 | HvMTP11.2 | 112509.A0A287G628 | ZT_dimer domain-containing protein |
| kmeans | 2 | Green | 18 | HvMTP12.1 | 112509.A0A287HEV8 | Uncharacterized protein |
| kmeans | 2 | Green | 18 | HvMTP5.1 | 112509.A0A287VA94 | Uncharacterized protein |
| kmeans | 2 | Green | 18 | HvMTP6.1 | 112509.A0A287NYW1 | ZT_dimer domain-containing protein |
| kmeans | 2 | Green | 18 | HvMTP7.1 | 112509.A0A287I953 | Uncharacterized protein |
| kmeans | 2 | Green | 18 | HvMTP8.2 | 112509.A0A287PDY3 | ZT_dimer domain-containing protein |
| kmeans | 2 | Green | 18 | M0UQC6 | 112509.M0UQC6 | Uncharacterized protein; Belongs to the TUB family. |
| kmeans | 2 | Green | 18 | M0YFH9 | 112509.M0YFH9 | Uncharacterized protein; Belongs to the TUB family. |
| kmeans | 3 | Blue | 5 | A0A287IX62 | 112509.A0A287IX62 | Fn3_like domain-containing protein |
| kmeans | 3 | Blue | 5 | A0A287MXD2 | 112509.A0A287MXD2 | NusB domain-containing protein |
| kmeans | 3 | Blue | 5 | A0A287P1G3 | 112509.A0A287P1G3 | Guanylate kinase-like domain-containing protein |
| kmeans | 3 | Blue | 5 | A0A287SB53 | 112509.A0A287SB53 | Uncharacterized protein |
| kmeans | 3 | Blue | 5 | HvMTP8.1 | 112509.A0A287UU57 | Uncharacterized protein |
