# Supplementary material for: Metal tolerance gene family in barley: an in silico comprehensive analysis
Source: J Appl Genet. 2022 Dec 31;64(2):197–215. doi: 10.1007/s13353-022-00744-6 (PMC10076399; doi:10.1007/s13353-022-00744-6)
Supplement: Supplementary file 2 — Supplementary file2 (DOCX 2449 KB) [file 13353_2022_744_MOESM2_ESM.docx]

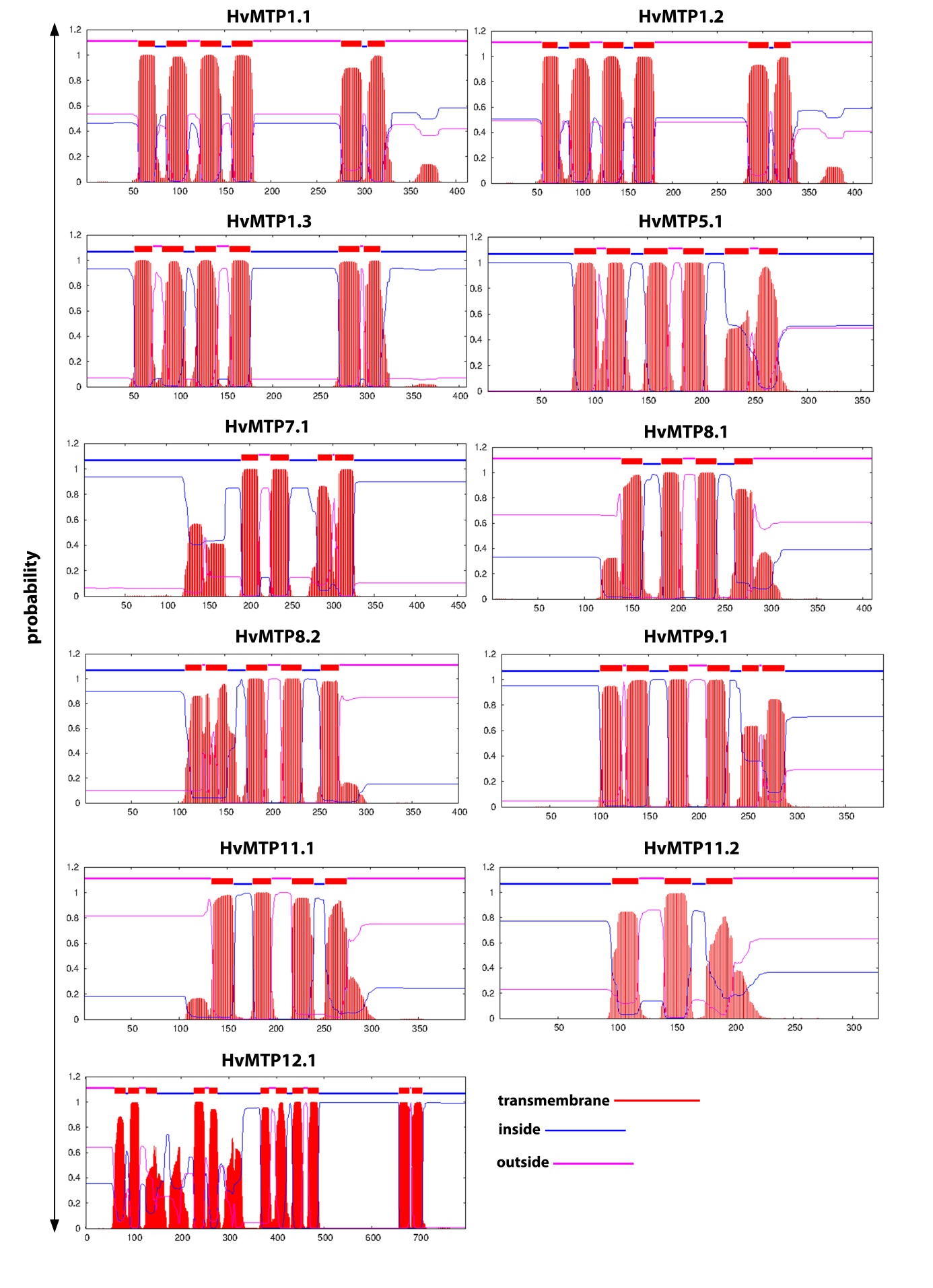


Supplementary figure S1. Prediction of transmembrane domains in HvMTP proteins.


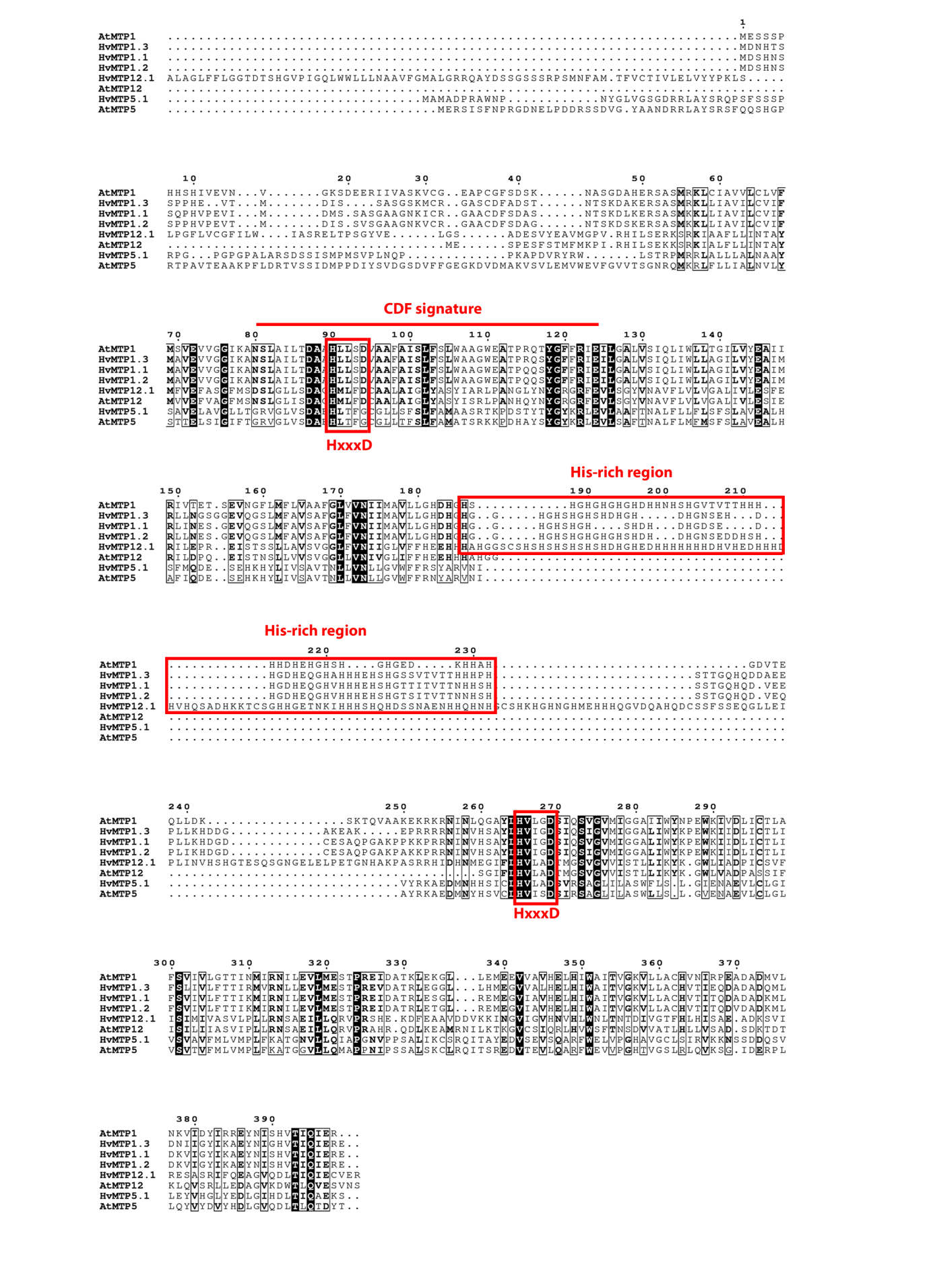


Supplementary Figure S2. The consensus sequence HxxxD, His-rich region, and CDF-signature in the HvMTP - Zn-CDFs (groups 1, 5, and 12) protein sequences.


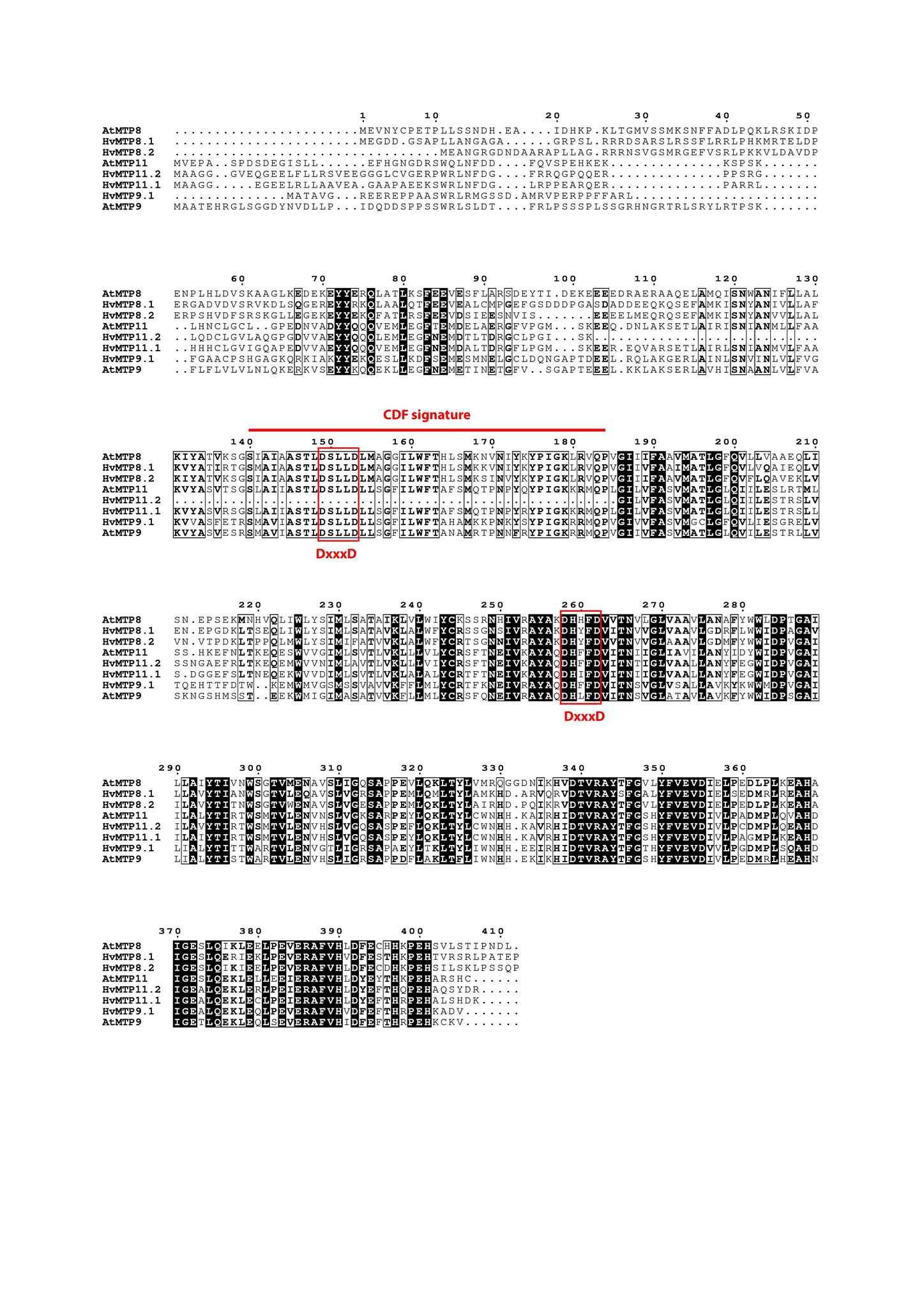


Supplementary Figure S3. The consensus sequence DxxxD, and CDF-signature in the HvMTP- Mn-CDFs (groups 8 and 9) protein sequences.


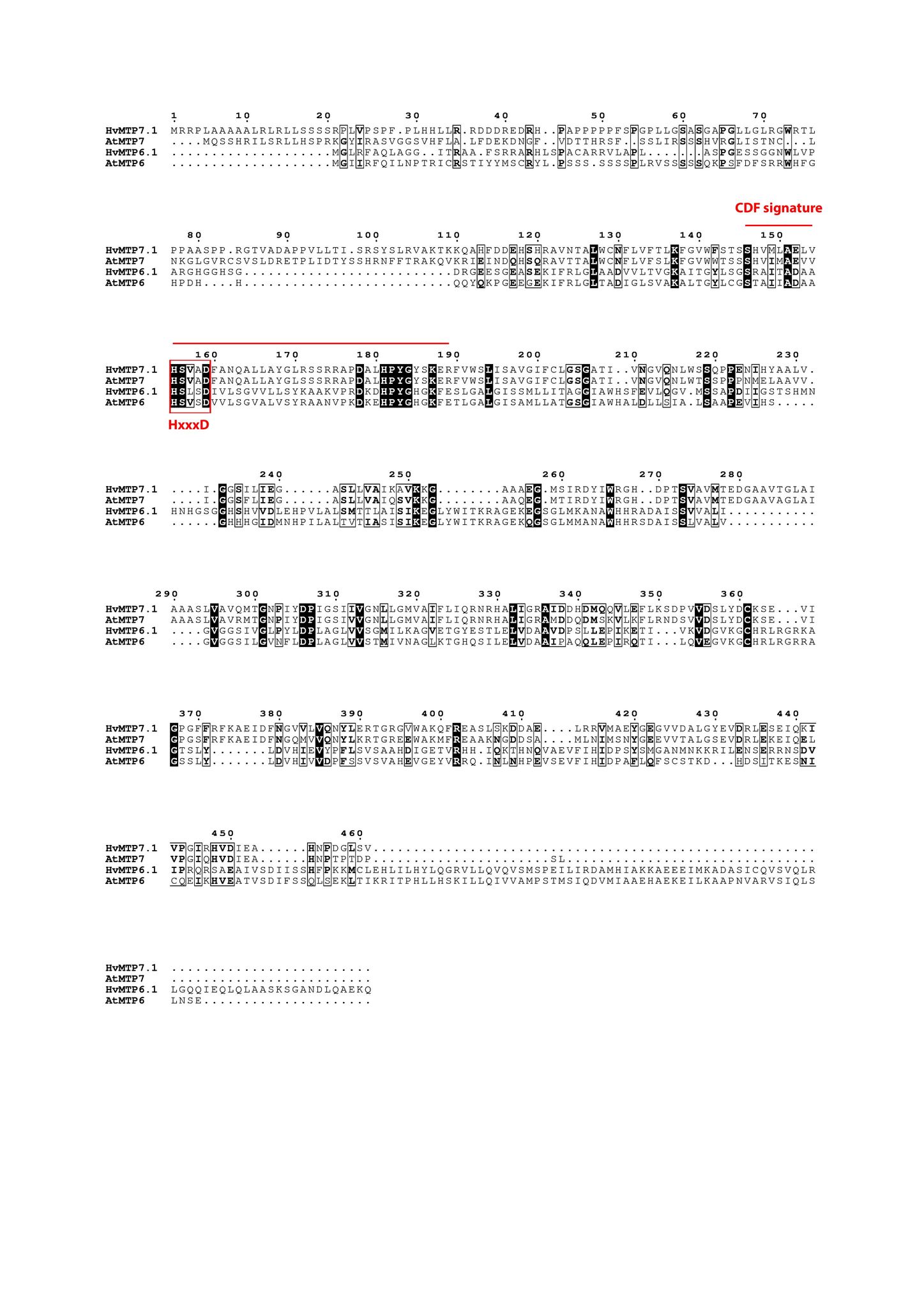


Supplementary Figure S4. The consensus sequence HxxxD, and CDF-signature in the HvMTP- Fe/Zn-CDFs (groups 6 and 7) protein sequences.


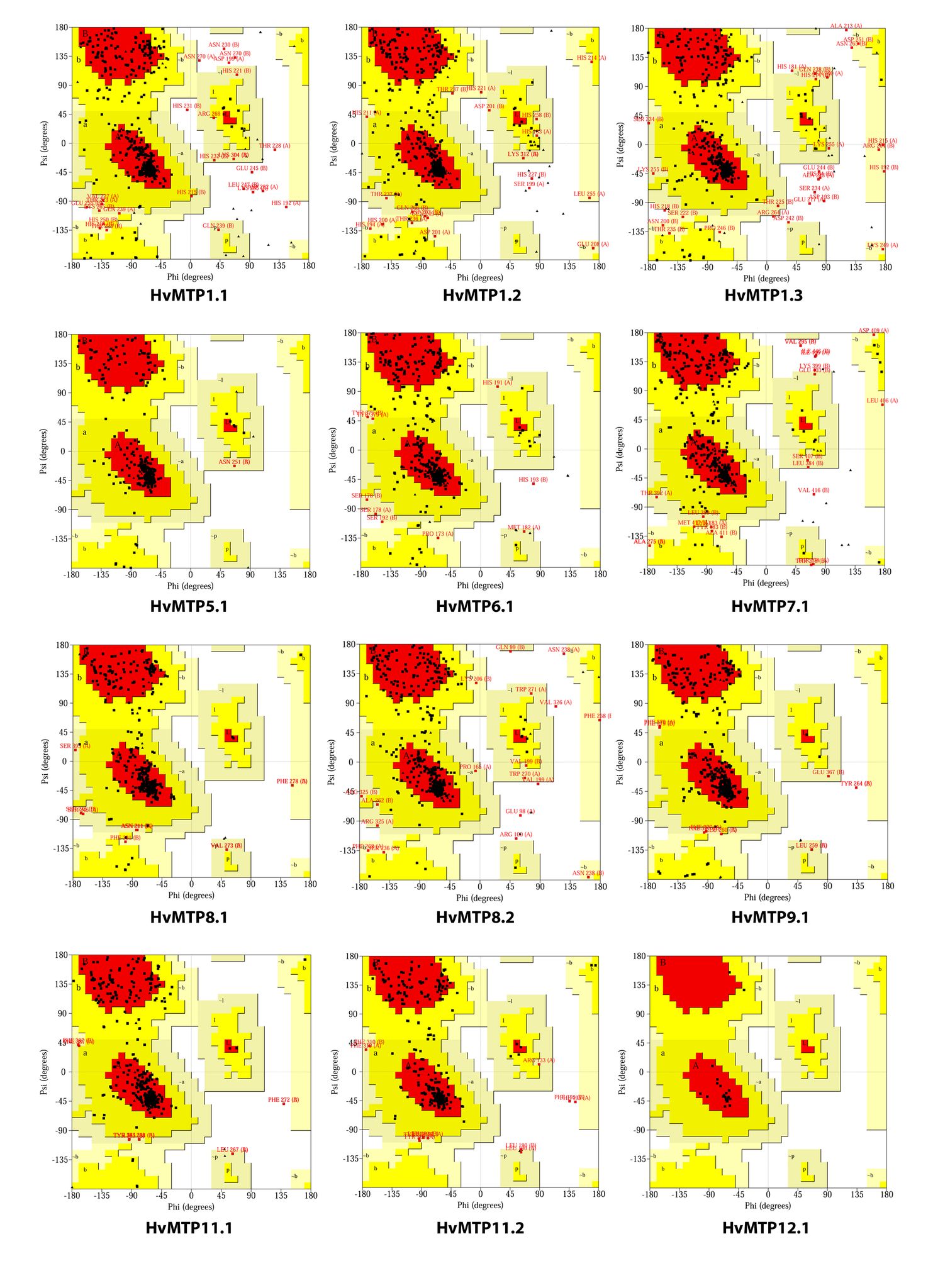


Supplementary Figure S5. Ramachandran plot analysis of homology models of HvMTP proteins.
